# Supplementary material for: The Healthy Smoker Paradox: Socioeconomic status as a fundamental cause of reversed anemia risk among Yemeni youth
Source: PLoS One. 2026 Apr 30;21(4):e0348146. doi: 10.1371/journal.pone.0348146 (PMC13132244; doi:10.1371/journal.pone.0348146)
Supplement: S1 Table — (DOCX) [file pone.0348146.s001.docx]

# Supporting TABLE S1

## COMPREHENSIVE BASELINE CHARACTERISTICS BY SMOKING STATUS

(N=600 Yemeni University Students)

| Characteristic | Overall (N=600) | Smokers (n=144) | Non-Smokers (n=456) | p-value |
| --- | --- | --- | --- | --- |
| === DEMOGRAPHIC CHARACTERISTICS === |  |  |  |  |
| Age (years) | 21.8 ± 2.3 | 22.1 ± 2.4 | 21.7 ± 2.2 | 0.087 |
| Gender |  |  |  | 0.215 |
| Male | 350 (58.3%) | 90 (62.5%) | 260 (56.8%) |  |
| Female | 250 (41.7%) | 54 (37.5%) | 196 (43.2%) |  |
| Place of Residence |  |  |  | 0.342 |
| Urban | 380 (63.3%) | 96 (66.7%) | 284 (62.3%) |  |
| Rural | 220 (36.7%) | 48 (33.3%) | 172 (37.7%) |  |
| === ACADEMIC CHARACTERISTICS === |  |  |  |  |
| University |  |  |  | 0.567 |
| UST-Aden | 200 (33.3%) | 52 (36.1%) | 148 (32.5%) |  |
| University of Lahej | 200 (33.3%) | 46 (31.9%) | 154 (33.8%) |  |
| AGIU-Al-Dhale | 200 (33.3%) | 46 (31.9%) | 154 (33.8%) |  |
| Faculty |  |  |  | 0.089 |
| Medical Sciences | 240 (40.0%) | 52 (36.1%) | 188 (41.2%) |  |
| Engineering | 180 (30.0%) | 54 (37.5%) | 126 (27.6%) |  |
| Humanities | 180 (30.0%) | 38 (26.4%) | 142 (31.1%) |  |
| === SOCIOECONOMIC CHARACTERISTICS === |  |  |  |  |
| Parental Education (University+) | 240 (40.0%) | 72 (50.0%) | 168 (36.8%) | 0.004 |
| Household Asset Index (0-15 scale) | 8.2 ± 3.1 | 9.8 ± 2.8 | 7.7 ± 3.0 | <0.001 |
| High Asset Index (>10 items) | 180 (30.0%) | 60 (41.7%) | 120 (26.3%) | <0.001 |
| Food Insecure | 150 (25.0%) | 24 (16.7%) | 126 (27.6%) | 0.008 |
| Household with Car | 240 (40.0%) | 72 (50.0%) | 168 (36.8%) | 0.004 |
| Internet Access at Home | 300 (50.0%) | 96 (66.7%) | 204 (44.7%) | <0.001 |
| === SMOKING CHARACTERISTICS (SMOKERS ONLY) === |  |  |  |  |
| Smoking Duration (years) | - | 3.2 ± 1.8 | - | - |
| Cigarettes per Day | - | 8.4 ± 5.1 | - | - |
| Smoking Intensity Categories |  |  |  |  |
| Light (1-5 cig/day) | - | 48 (33.3%) | - |  |
| Moderate (6-10 cig/day) | - | 60 (41.7%) | - |  |
| Heavy (11-20 cig/day) | - | 30 (20.8%) | - |  |
| Very Heavy (>20 cig/day) | - | 6 (4.2%) | - |  |
| Age Started Smoking (years) | - | 18.2 ± 2.1 | - | - |
| Multiple Tobacco Product Use | - | 36 (25.0%) | - | - |
| === ANTHROPOMETRIC MEASUREMENTS === |  |  |  |  |
| BMI (kg/m²) | 23.2 ± 3.1 | 23.5 ± 3.3 | 23.1 ± 3.0 | 0.189 |
| BMI Categories |  |  |  | 0.423 |
| Underweight (<18.5) | 60 (10.0%) | 12 (8.3%) | 48 (10.5%) |  |
| Normal (18.5-24.9) | 360 (60.0%) | 84 (58.3%) | 276 (60.5%) |  |
| Overweight (25-29.9) | 150 (25.0%) | 42 (29.2%) | 108 (23.7%) |  |
| Obese (≥30) | 30 (5.0%) | 6 (4.2%) | 24 (5.3%) |  |
| === HEMATOLOGICAL PARAMETERS (CONTINUOUS) === |  |  |  |  |
| Hemoglobin (g/dL) | 13.8 ± 1.6 | 14.9 ± 1.2 | 13.4 ± 1.5 | <0.001 |
| MCHC (g/dL) | 32.5 ± 1.3 | 33.1 ± 1.0 | 32.3 ± 1.3 | <0.001 |
| MCV (fL) | 86.2 ± 5.4 | 87.1 ± 4.8 | 85.9 ± 5.6 | 0.023 |
| MCH (pg) | 28.9 ± 2.1 | 29.4 ± 1.8 | 28.7 ± 2.2 | 0.001 |
| Platelets (×10³/μL) | 298 ± 78 | 291 ± 74 | 301 ± 79 | 0.167 |
| White Blood Cells (×10³/μL) | 6.8 ± 1.9 | 7.1 ± 1.8 | 6.7 ± 1.9 | 0.034 |
| PT (seconds) | 12.8 ± 1.2 | 12.6 ± 1.1 | 12.9 ± 1.2 | 0.018 |
| APTT (seconds) | 33.2 ± 4.1 | 32.8 ± 3.9 | 33.4 ± 4.2 | 0.134 |
| === HEMATOLOGICAL ABNORMALITIES (CATEGORICAL) === |  |  |  |  |
| Anemia (Hb <13M/<12F) | 164 (27.3%) | 6 (4.2%) | 158 (34.2%) | <0.001 |
| Abnormal MCHC (<32 g/dL) | 168 (28.0%) | 18 (12.5%) | 150 (32.9%) | <0.001 |
| Microcytosis (MCV <80 fL) | 42 (7.0%) | 6 (4.2%) | 36 (7.9%) | 0.127 |
| Thrombocytopenia (PLT <150) | 34 (5.7%) | 6 (4.2%) | 28 (6.1%) | 0.389 |
| Abnormal PT (>14 seconds) | 274 (45.7%) | 78 (54.2%) | 196 (42.5%) | 0.012 |
| Abnormal APTT (>38 seconds) | 342 (57.0%) | 96 (66.7%) | 246 (53.5%) | 0.005 |
| === OTHER BEHAVIORAL CHARACTERISTICS === |  |  |  |  |
| Khat Chewing Frequency |  |  |  | 0.315 |
| Never | 240 (40.0%) | 54 (37.5%) | 186 (40.8%) |  |
| Occasionally | 180 (30.0%) | 42 (29.2%) | 138 (30.3%) |  |
| Weekly | 120 (20.0%) | 36 (25.0%) | 84 (18.4%) |  |
| Daily | 60 (10.0%) | 12 (8.3%) | 48 (10.5%) |  |
| Sleep Duration |  |  |  | 0.078 |
| <7 hours | 312 (52.0%) | 84 (58.3%) | 228 (50.0%) |  |
| 7-8 hours | 228 (38.0%) | 48 (33.3%) | 180 (39.5%) |  |
| >8 hours | 60 (10.0%) | 12 (8.3%) | 48 (10.5%) |  |
| Physical Activity (≥3 days/week) | 180 (30.0%) | 54 (37.5%) | 126 (27.6%) | 0.023 |
| === DIETARY CHARACTERISTICS === |  |  |  |  |
| Dietary Diversity Score (0-9) | 5.2 ± 1.8 | 6.1 ± 1.5 | 4.9 ± 1.8 | <0.001 |
| Low Dietary Diversity (<4 groups) | 150 (25.0%) | 18 (12.5%) | 132 (28.9%) | <0.001 |
| Regular Breakfast Consumption | 300 (50.0%) | 90 (62.5%) | 210 (46.1%) | 0.001 |
| Nutritional Supplement Use | 90 (15.0%) | 30 (20.8%) | 60 (13.2%) | 0.024 |
