# Supplementary material for: The Healthy Smoker Paradox: Socioeconomic status as a fundamental cause of reversed anemia risk among Yemeni youth
Source: PLoS One. 2026 Apr 30;21(4):e0348146. doi: 10.1371/journal.pone.0348146 (PMC13132244; doi:10.1371/journal.pone.0348146)
Supplement: S2 Table — (DOCX) [file pone.0348146.s002.docx]

# Supporting TABLE S2 – COMPLETE MULTIVARIABLE LOGISTIC REGRESSION RESULTS

## OUTCOME: ABNORMAL HEMOGLOBIN (Anemia)

| Predictor | β-coeff | SE | Wald | p-value | aOR | 95% CI Lower | 95% CI Upper |
| --- | --- | --- | --- | --- | --- | --- | --- |
| Intercept | -2.345 | 0.891 | 6.93 | 0.009 | - | - | - |
| Non-smoker | 2.421 | 0.589 | 16.90 | <0.001 | 11.25 | 3.45 | 36.70 |
| Age | -0.134 | 0.104 | 1.66 | 0.198 | 0.87 | 0.71 | 1.07 |
| Male | 1.892 | 0.342 | 30.58 | <0.001 | 6.63 | 3.39 | 12.98 |
| BMI | -0.067 | 0.055 | 1.48 | 0.223 | 0.94 | 0.84 | 1.04 |
| University of Lahej | 0.234 | 0.301 | 0.60 | 0.437 | 1.26 | 0.70 | 2.28 |
| AGIU-Al-Dhale | 0.189 | 0.315 | 0.36 | 0.548 | 1.21 | 0.65 | 2.24 |

Model Fit Statistics:

- Log-likelihood: -285.34

- AIC: 582.68

- Cox & Snell R²: 0.189

- Nagelkerke R²: 0.284

- Hosmer-Lemeshow: χ²=7.23, p=0.512

## OUTCOME: ABNORMAL MCHC (Hypochromia)

| Predictor | β-coeff | SE | Wald | p-value | aOR | 95% CI Lower | 95% CI Upper |
| --- | --- | --- | --- | --- | --- | --- | --- |
| Intercept | -1.892 | 0.745 | 6.45 | 0.011 | - | - | - |
| Non-smoker | 1.227 | 0.388 | 10.01 | 0.002 | 3.41 | 1.58 | 7.35 |
| Age | -0.089 | 0.082 | 1.18 | 0.277 | 0.91 | 0.78 | 1.07 |
| Male | 0.784 | 0.267 | 8.63 | 0.003 | 2.19 | 1.30 | 3.70 |
| BMI | -0.045 | 0.043 | 1.09 | 0.295 | 0.96 | 0.88 | 1.04 |
| University of Lahej | 0.156 | 0.245 | 0.41 | 0.524 | 1.17 | 0.72 | 1.89 |
| AGIU-Al-Dhale | 0.134 | 0.258 | 0.27 | 0.603 | 1.14 | 0.69 | 1.89 |

Model Fit Statistics:

- Log-likelihood: -312.56

- AIC: 637.12

- Cox & Snell R²: 0.078

- Nagelkerke R²: 0.112

- Hosmer-Lemeshow: χ²=5.89, p=0.659

## OUTCOME: ABNORMAL PLATELETS (Thrombocytopenia)

| Predictor | β-coeff | SE | Wald | p-value | aOR | 95% CI Lower | 95% CI Upper |
| --- | --- | --- | --- | --- | --- | --- | --- |
| Intercept | -3.456 | 1.234 | 7.85 | 0.005 | - | - | - |
| <7 hours | 0.456 | 0.512 | 0.79 | 0.373 | 1.58 | 0.58 | 4.30 |
| 8-11 hours | 1.416 | 0.475 | 8.89 | 0.003 | 4.12 | 1.62 | 10.49 |
| Age | 0.088 | 0.132 | 0.44 | 0.505 | 1.09 | 0.84 | 1.41 |
| Male | -0.215 | 0.424 | 0.26 | 0.612 | 0.81 | 0.35 | 1.86 |
| BMI | -0.042 | 0.070 | 0.36 | 0.549 | 0.96 | 0.84 | 1.10 |
| University of Lahej | 0.189 | 0.445 | 0.18 | 0.671 | 1.21 | 0.51 | 2.89 |
| AGIU-Al-Dhale | 0.156 | 0.458 | 0.12 | 0.733 | 1.17 | 0.48 | 2.86 |

Model Fit Statistics:

- Log-likelihood: -145.23

- AIC: 304.46

- Cox & Snell R²: 0.045

- Nagelkerke R²: 0.089

- Hosmer-Lemeshow: χ²=4.56, p=0.803

## OUTCOME: ABNORMAL PT (Coagulation)

| Predictor | β-coeff | SE | Wald | p-value | aOR | 95% CI Lower | 95% CI Upper |
| --- | --- | --- | --- | --- | --- | --- | --- |
| Intercept | -1.234 | 0.678 | 3.31 | 0.069 | - | - | - |
| Occasionally | 0.456 | 0.345 | 1.75 | 0.186 | 1.58 | 0.80 | 3.11 |
| Weekly | 0.837 | 0.402 | 4.34 | 0.037 | 2.31 | 1.05 | 5.08 |
| Daily | 0.678 | 0.423 | 2.57 | 0.109 | 1.97 | 0.86 | 4.51 |
| Age | 0.102 | 0.089 | 1.31 | 0.252 | 1.11 | 0.93 | 1.32 |
| Male | 0.456 | 0.301 | 2.29 | 0.129 | 1.58 | 0.88 | 2.84 |
| BMI | 0.034 | 0.047 | 0.52 | 0.471 | 1.03 | 0.94 | 1.13 |
| University of Lahej | 0.189 | 0.278 | 0.46 | 0.497 | 1.21 | 0.70 | 2.09 |
| AGIU-Al-Dhale | 0.167 | 0.291 | 0.33 | 0.566 | 1.18 | 0.67 | 2.09 |

Model Fit Statistics:

- Log-likelihood: -398.45

- AIC: 814.90

- Cox & Snell R²: 0.067

- Nagelkerke R²: 0.090

- Hosmer-Lemeshow: χ²=6.78, p=0.561

## OUTCOME: ABNORMAL APTT (Coagulation)

| Predictor | β-coeff | SE | Wald | p-value | aOR | 95% CI Lower | 95% CI Upper |
| --- | --- | --- | --- | --- | --- | --- | --- |
| Intercept | -0.891 | 0.612 | 2.12 | 0.145 | - | - | - |
| Occasionally | 0.789 | 0.378 | 4.35 | 0.037 | 2.20 | 1.05 | 4.62 |
| Weekly | 1.330 | 0.445 | 8.93 | 0.003 | 3.78 | 1.55 | 9.22 |
| Daily | 1.145 | 0.467 | 6.01 | 0.014 | 3.14 | 1.26 | 7.85 |
| Age | -0.056 | 0.094 | 0.35 | 0.552 | 0.95 | 0.79 | 1.14 |
| Male | 0.289 | 0.318 | 0.83 | 0.363 | 1.34 | 0.72 | 2.49 |
| BMI | 0.021 | 0.050 | 0.18 | 0.675 | 1.02 | 0.93 | 1.12 |
| University of Lahej | 0.134 | 0.301 | 0.20 | 0.657 | 1.14 | 0.63 | 2.06 |
| AGIU-Al-Dhale | 0.156 | 0.315 | 0.25 | 0.620 | 1.17 | 0.63 | 2.17 |

Model Fit Statistics:

- Log-likelihood: -378.90

- AIC: 775.80

- Cox & Snell R²: 0.089

- Nagelkerke R²: 0.119

- Hosmer-Lemeshow: χ²=5.34, p=0.721

Notes: aOR = adjusted Odds Ratio; CI = Confidence Interval; SE = Standard Error

All models adjusted for age, gender, BMI, and university site.

Reference categories: Smoking status = Smoker; Gender = Female; University = UST-Aden
