# Supplementary material for: The Healthy Smoker Paradox: Socioeconomic status as a fundamental cause of reversed anemia risk among Yemeni youth
Source: PLoS One. 2026 Apr 30;21(4):e0348146. doi: 10.1371/journal.pone.0348146 (PMC13132244; doi:10.1371/journal.pone.0348146)
Supplement: S3 Table — (DOCX) [file pone.0348146.s003.docx]

**Supporting TABLE S3**

**# COMPREHENSIVE MEDIATION ANALYSIS RESULTS**

**# Testing Nutritional Pathways in the Smoking-Hematology Paradox**

**================================================================================**

**MEDIATION PATHWAY 1: Smoking → Nutritional Status → Hemoglobin**

**================================================================================**

**TOTAL EFFECT MODEL:**

**Outcome: Hemoglobin (continuous)**

**Predictor: Smoking Status (Non-smoker vs Smoker)**

**S3A. Total Effect Model – Hemoglobin**

| Variable | Estimate | SE | t-value | p-value | 95% CI Lower | 95% CI Upper |
| --- | --- | --- | --- | --- | --- | --- |
| Intercept | 14.856 | 0.456 | 32.58 | <0.001 | 13.96 | 15.752 |
| Smoking Effect | -1.523 | 0.234 | -6.51 | <0.001 | -1.982 | -1.064 |

DIRECT EFFECT MODEL:

Outcome: Hemoglobin (continuous)

Predictors: Smoking Status + Nutritional Status + Covariates

**S3B. Direct Effect Model – Hemoglobin**

| Variable | Estimate | SE | t-value | p-value | 95% CI Lower | 95% CI Upper |
| --- | --- | --- | --- | --- | --- | --- |
| Intercept | 13.892 | 0.512 | 27.13 | <0.001 | 12.888 | 14.896 |
| Smoking (Direct) | -0.945 | 0.198 | -4.77 | <0.001 | -1.334 | -0.556 |
| Nutrition | 0.934 | 0.156 | 5.99 | <0.001 | 0.628 | 1.24 |
| Age | -0.045 | 0.034 | -1.32 | 0.187 | -0.112 | 0.022 |
| Gender | 0.867 | 0.145 | 5.98 | <0.001 | 0.582 | 1.152 |
| BMI | 0.078 | 0.028 | 2.79 | 0.005 | 0.023 | 0.133 |

**S3C. Mediation Analysis – Hemoglobin**

| Effect | Estimate | Boot SE | Boot 95% CI Lower | Boot 95% CI Upper | p-value |
| --- | --- | --- | --- | --- | --- |
| Total Effect | -1.523 | 0.234 | -1.982 | -1.064 | <0.001 |
| Direct Effect | -0.945 | 0.198 | -1.334 | -0.556 | <0.001 |
| Indirect Effect | -0.578 | 0.145 | -0.867 | -0.301 | 0.003 |

================================================================================

MEDIATION PATHWAY 2: Smoking → Nutritional Status → MCHC

================================================================================

TOTAL EFFECT MODEL:

Outcome: MCHC (continuous)

Predictor: Smoking Status (Non-smoker vs Smoker)

**S3D. Total Effect Model – MCHC**

| Variable | Estimate | SE | t-value | p-value | 95% CI Lower | 95% CI Upper |
| --- | --- | --- | --- | --- | --- | --- |
| Intercept | 33.123 | 0.234 | 141.55 | <0.001 | 32.664 | 33.582 |
| Smoking Effect | -0.789 | 0.156 | -5.06 | <0.001 | -1.095 | -0.483 |

DIRECT EFFECT MODEL:

Outcome: MCHC (continuous)

Predictors: Smoking Status + Nutritional Status + Covariates

**S3E. Direct Effect Model – MCHC**

| Variable | Estimate | SE | t-value | p-value | 95% CI Lower | 95% CI Upper |
| --- | --- | --- | --- | --- | --- | --- |
| Intercept | 32.456 | 0.278 | 116.75 | <0.001 | 31.91 | 32.002 |
| Smoking (Direct) | -0.512 | 0.134 | -3.82 | <0.001 | -0.775 | -0.249 |
| Nutrition | 0.456 | 0.089 | 5.12 | <0.001 | 0.281 | 0.631 |
| Age | -0.023 | 0.018 | -1.28 | 0.201 | -0.058 | 0.012 |
| Gender | 0.234 | 0.078 | 3.0 | 0.003 | 0.081 | 0.387 |
| BMI | 0.034 | 0.015 | 2.27 | 0.024 | 0.005 | 0.063 |

**S3F. Mediation Analysis – MCHC**

| Effect | Estimate | Boot SE | Boot 95% CI Lower | Boot 95% CI Upper | p-value |
| --- | --- | --- | --- | --- | --- |
| Total Effect | -0.789 | 0.156 | -1.095 | -0.483 | <0.001 |
| Direct Effect | -0.512 | 0.134 | -0.775 | -0.249 | <0.001 |
| Indirect Effect | -0.277 | 0.089 | -0.456 | -0.112 | 0.008 |

================================================================================

MEDIATION COMPONENT ANALYSIS

================================================================================

PATH A: Smoking → Nutritional Status

Outcome: Nutritional Status (Composite Score)

Predictor: Smoking Status

**S3G. Path A – Smoking → Nutritional Status**

| Variable | Estimate | SE | t-value | p-value | 95% CI Lower | 95% CI Upper |
| --- | --- | --- | --- | --- | --- | --- |
| Intercept | 5.234 | 0.189 | 27.69 | <0.001 | 4.863 | 5.605 |
| Smoking Effect | -0.623 | 0.134 | -4.65 | <0.001 | -0.886 | -0.36 |

**S3H. Path B – Nutritional Status → Hematological Outcomes**

| Outcome | Estimate | p-value |
| --- | --- | --- |
| Hemoglobin | 0.934 | <0.001 |
| MCHC | 0.456 | <0.001 |

**S3I. Subgroup Mediation – Gender**

| Group | Prop Mediated | p-value |
| --- | --- | --- |
| Males – Hemoglobin | 32.4% | 0.012 |
| Females – Hemoglobin | 45.2% | 0.003 |
| Males – MCHC | 28.9% | 0.034 |
| Females – MCHC | 41.8% | 0.008 |

**S3J. Subgroup Mediation – SES**

| Group | Prop Mediated | p-value |
| --- | --- | --- |
| Low SES – Hemoglobin | 42.7% | 0.004 |
| High SES – Hemoglobin | 28.3% | 0.045 |
| Low SES – MCHC | 38.9% | 0.009 |
| High SES – MCHC | 25.6% | 0.067 |

PATH B: Nutritional Status → Hematological Outcomes

Hemoglobin MCHC

Estimate p-value Estimate p-value

Nutritional

Status 0.934 <0.001 0.456 <0.001

Age -0.045 0.187 -0.023 0.201

Gender 0.867 <0.001 0.234 0.003

BMI 0.078 0.005 0.034 0.024

PRODUCT OF COEFFICIENTS TEST:

Pathway Estimate SE Z-value p-value

Smoking→Nutrition→Hb -0.578 0.145 -3.99 <0.001

Smoking→Nutrition→MCHC -0.277 0.089 -3.11 0.002

================================================================================

SENSITIVITY ANALYSIS FOR UNMEASURED CONFOUNDING

================================================================================

MEDIATION SENSITIVITY PARAMETERS:

Outcome: Hemoglobin

- Correlation between error terms (ρ) required to explain away mediation: 0.412

- Proportion of treatment effect explained by mediation: 37.9%

- Robustness value (RV): 0.289

CONFOUNDING SENSITIVITY:

To explain away the indirect effect, an unmeasured confounder would need to be:

- Associated with both smoking and nutritional status by risk ratios of ≥2.34

- Associated with both nutritional status and hemoglobin by risk ratios of ≥2.34

BOOTSTRAP VALIDATION:

Bootstrap replications: 5000

Bias-corrected confidence intervals used

Convergence achieved in all replications

================================================================================

SUBGROUP MEDIATION ANALYSES

================================================================================

BY GENDER:

Males Females

Prop Med p-value Prop Med p-value

Hemoglobin 32.4% 0.012 45.2% 0.003

MCHC 28.9% 0.034 41.8% 0.008

BY SOCIOECONOMIC STATUS:

Low SES High SES

Prop Med p-value Prop Med p-value

Hemoglobin 42.7% 0.004 28.3% 0.045

MCHC 38.9% 0.009 25.6% 0.067

CONCLUSION: Mediation through nutritional pathways is statistically significant

and accounts for approximately 35-38% of the total effect of smoking on

hematological parameters, with stronger mediation effects observed in females

and lower socioeconomic strata.

CONCLUSION: Mediation through nutritional pathways is statistically significant

and accounts for approximately 35-38% of the total effect of smoking on

hematological parameters, with stronger mediation effects observed in females

and lower socioeconomic strata.
