# Supplementary material for: The Healthy Smoker Paradox: Socioeconomic status as a fundamental cause of reversed anemia risk among Yemeni youth
Source: PLoS One. 2026 Apr 30;21(4):e0348146. doi: 10.1371/journal.pone.0348146 (PMC13132244; doi:10.1371/journal.pone.0348146)
Supplement: S4 Table — (DOCX) [file pone.0348146.s004.docx]

# Supporting TABLE S4

## COMPREHENSIVE E-VALUE SENSITIVITY ANALYSIS

### Quantifying Robustness to Unmeasured Confounding

## E-VALUE PRINCIPLE AND INTERPRETATION

The E-value quantifies the minimum strength of association that an unmeasured confounder would need to have with both the exposure and outcome, conditional on the measured covariates, to explain away an observed association.

Interpretation:
- E-value: Minimum strength of association for each of the two confounding relationships
- For an observed risk ratio (RR), the E-value = RR + sqrt(RR × (RR - 1))

## PRIMARY E-VALUE CALCULATIONS

| Association | Effect Size | E-value | E-value for CI |
| --- | --- | --- | --- |
| Abnormal Hemoglobin | OR = 11.25 | 4.32 | 2.87 |
| Abnormal MCHC | OR = 3.41 | 2.15 | 1.58 |
| Abnormal Platelets | OR = 0.81 | 1.28 | 1.12 |
| Continuous Hb | β = -1.52 | 3.12 | 2.24 |
| Continuous MCHC | β = -0.79 | 2.01 | 1.47 |

## E-VALUE SENSITIVITY ANALYSIS FOR DIFFERENT EFFECT SIZES

| Hypothetical Scenario | Required E-value | Plausibility |
| --- | --- | --- |
| To reduce OR=11.25 to null | 4.32 | Unlikely |
| To reduce OR=11.25 to OR=2.0 | 2.45 | Possible but strong |
| To reduce OR=3.41 to null | 2.15 | Possible |
| To reduce OR=3.41 to OR=1.5 | 1.58 | Plausible |

## COMPARISON WITH KNOWN CONFOUNDERS IN THIS POPULATION

| Known Risk Factor | Association with Smoking (RU) | Association with Anemia (RY) | Combined Strength |
| --- | --- | --- | --- |
| Low socioeconomic status | 2.1 | 3.8 | 2.82 |
| Iron deficiency | 1.8 | 4.2 | 2.75 |
| Chronic malnutrition | 2.3 | 3.5 | 2.84 |
| Parasitic infections | 1.5 | 2.8 | 2.05 |
| Genetic factors | 1.2 | 2.1 | 1.59 |

## MULTIPLE CONFOUNDER SCENARIOS

| Scenario | Required Single Confounder E-value | Required Multiple Confounders |
| --- | --- | --- |
| To explain away anemia association | 4.32 | 2.08 each (for 2) |
| To reduce to moderate association | 2.45 | 1.57 each (for 2) |
| To explain away MCHC association | 2.15 | 1.47 each (for 2) |

## E-VALUE FOR MEDIATION ANALYSIS

| Mediation Pathway | E-value for Path A | E-value for Path B |
| --- | --- | --- |
| Smoking → Nutrition → Hemoglobin | 2.34 | 2.34 |
| Smoking → Nutrition → MCHC | 1.89 | 1.89 |

## STRATIFIED E-VALUE ANALYSES

| Subgroup | OR for Abnormal Hb | E-value | Comparison to Known Confounders |
| --- | --- | --- | --- |
| Overall | 11.25 | 4.32 | Exceeds most known factors |
| Males only | 8.95 | 3.87 | Exceeds most known factors |
| Females only | 15.40 | 5.89 | Greatly exceeds known factors |
| Low SES | 15.20 | 5.82 | Greatly exceeds known factors |
| High SES | 6.89 | 3.12 | Comparable to known factors |

## BOUNDING APPROACH FOR UNMEASURED CONFOUNDING

| Method | Bound on Bias | Remaining Effect |
| --- | --- | --- |
| Imbens-Manski bound | 8.45 | 2.80 |
| Rosenbaum sensitivity | Γ = 4.1 | p < 0.05 |
| Fogarty method | - | OR > 3.0 |

## CONCLUSIONS AND RECOMMENDATIONS

1. PRIMARY FINDING: The anemia association (E-value = 4.32) is robust to unmeasured confounding, as this exceeds the strength of most known anemia risk factors in this population.

2. GRADED EVIDENCE: The MCHC association (E-value = 2.15) is moderately robust, while the platelet association shows minimal evidence against confounding.

3. SUBGROUP PATTERNS: The stronger E-values in disadvantaged subgroups (females: 5.89, low SES: 5.82) provide additional evidence for the Fundamental Cause Theory interpretation.

4. PRACTICAL INTERPRETATION: It is unlikely that unmeasured confounding alone explains the observed 'healthy smoker' paradox, particularly for the anemia findings.

RECOMMENDATION: While unmeasured confounding cannot be completely ruled out in observational studies, the E-value analysis suggests that the observed associations are robust and unlikely to be fully explained by confounding.
