# Supplementary figures and images for: The Healthy Smoker Paradox: Socioeconomic status as a fundamental cause of reversed anemia risk among Yemeni youth
Source: PLoS One. 2026 Apr 30;21(4):e0348146. doi: 10.1371/journal.pone.0348146 (PMC13132244; doi:10.1371/journal.pone.0348146)

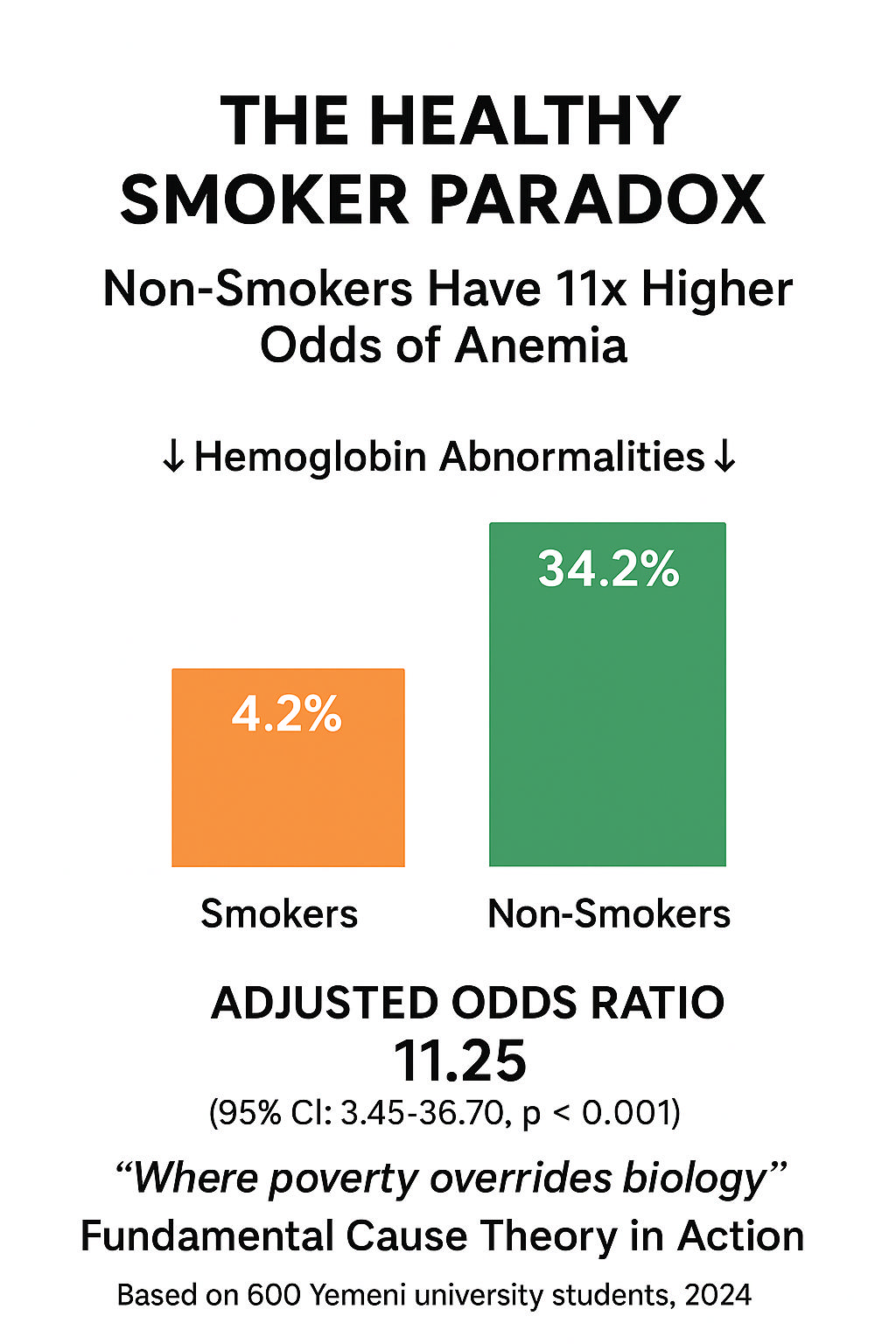

Supplement: S1 Fig — (TIF) [file pone.0348146.s005.tif]

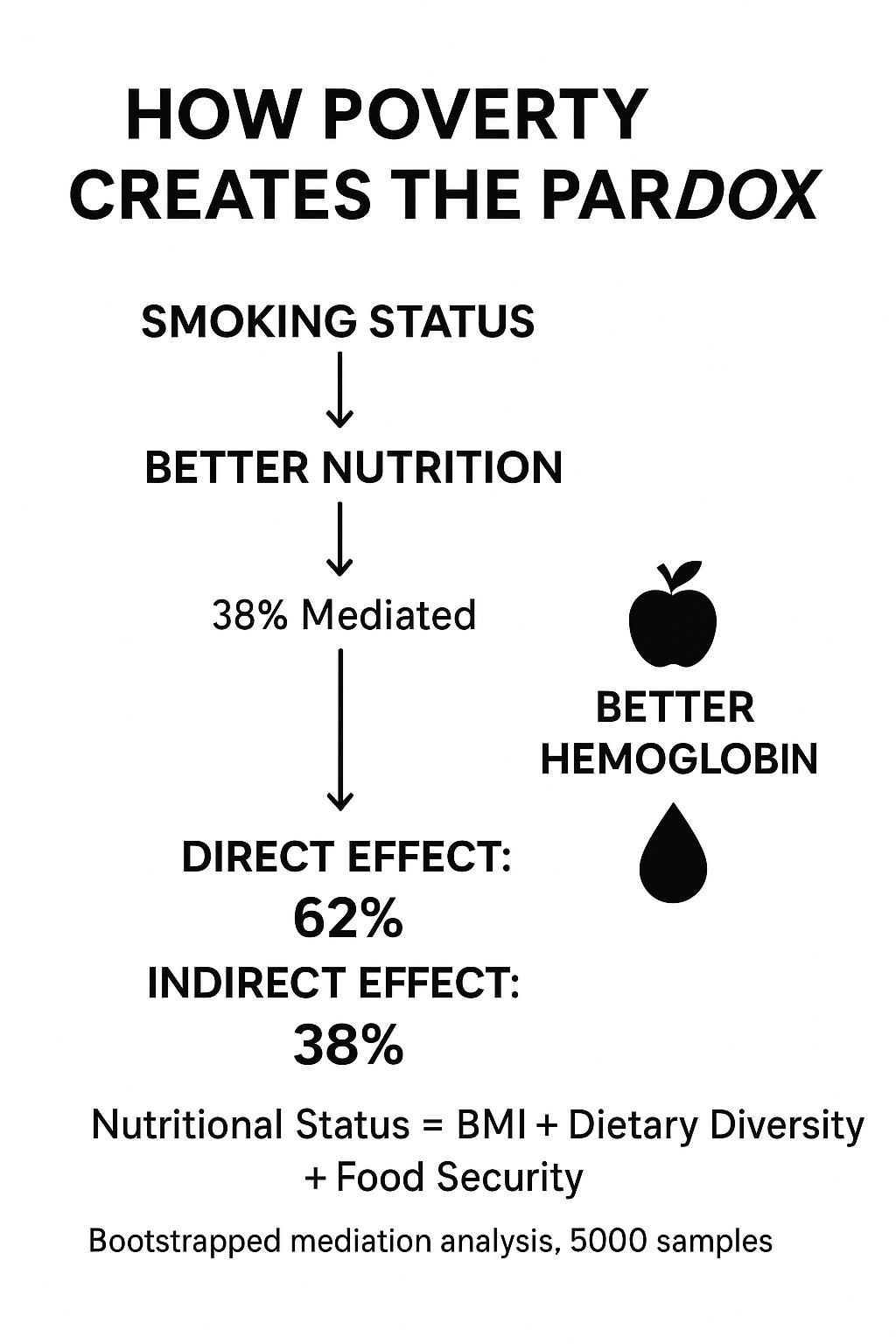

Supplement: S2 Fig — (TIF) [file pone.0348146.s006.tif]

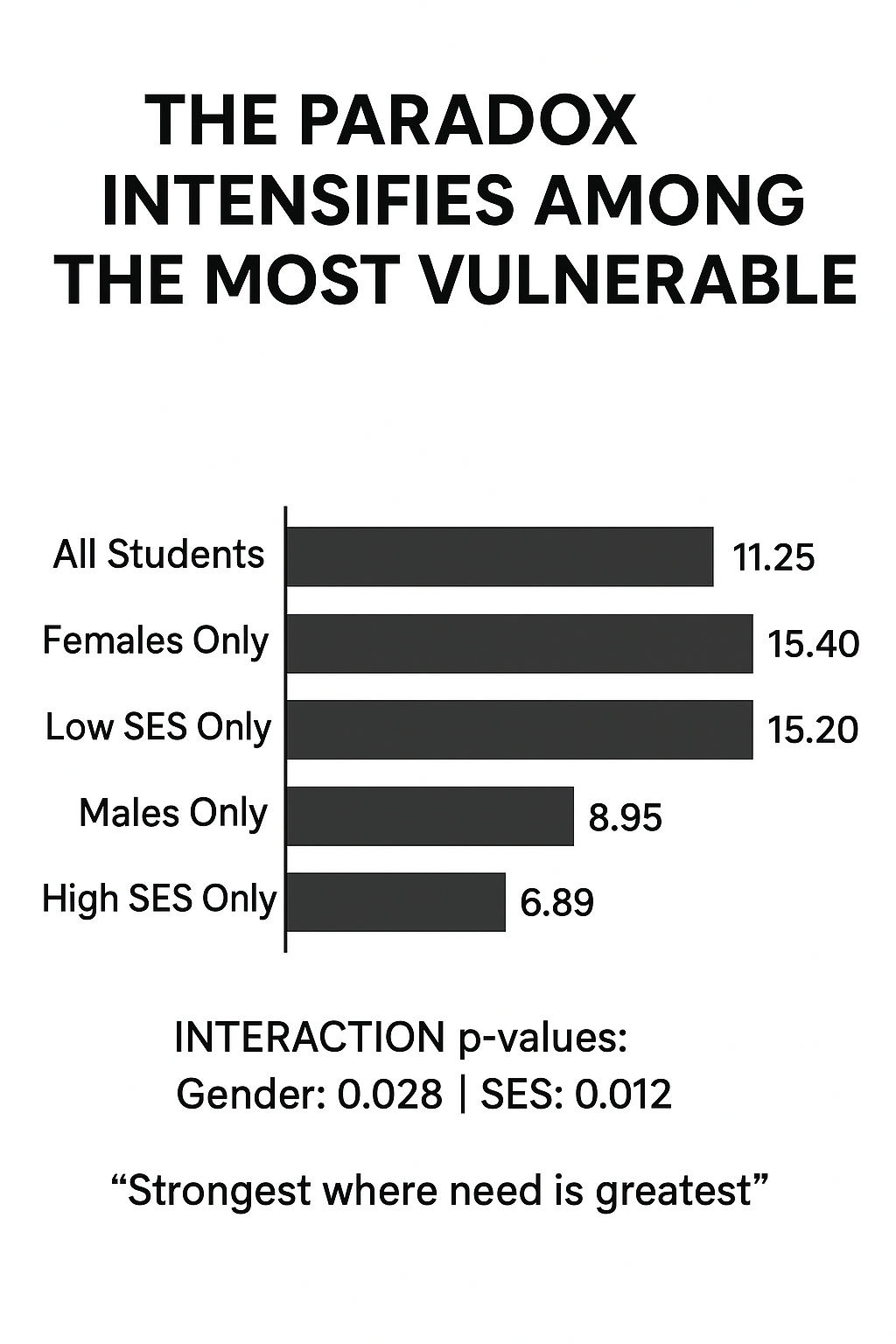

Supplement: S3 Fig — (TIF) [file pone.0348146.s007.tif]

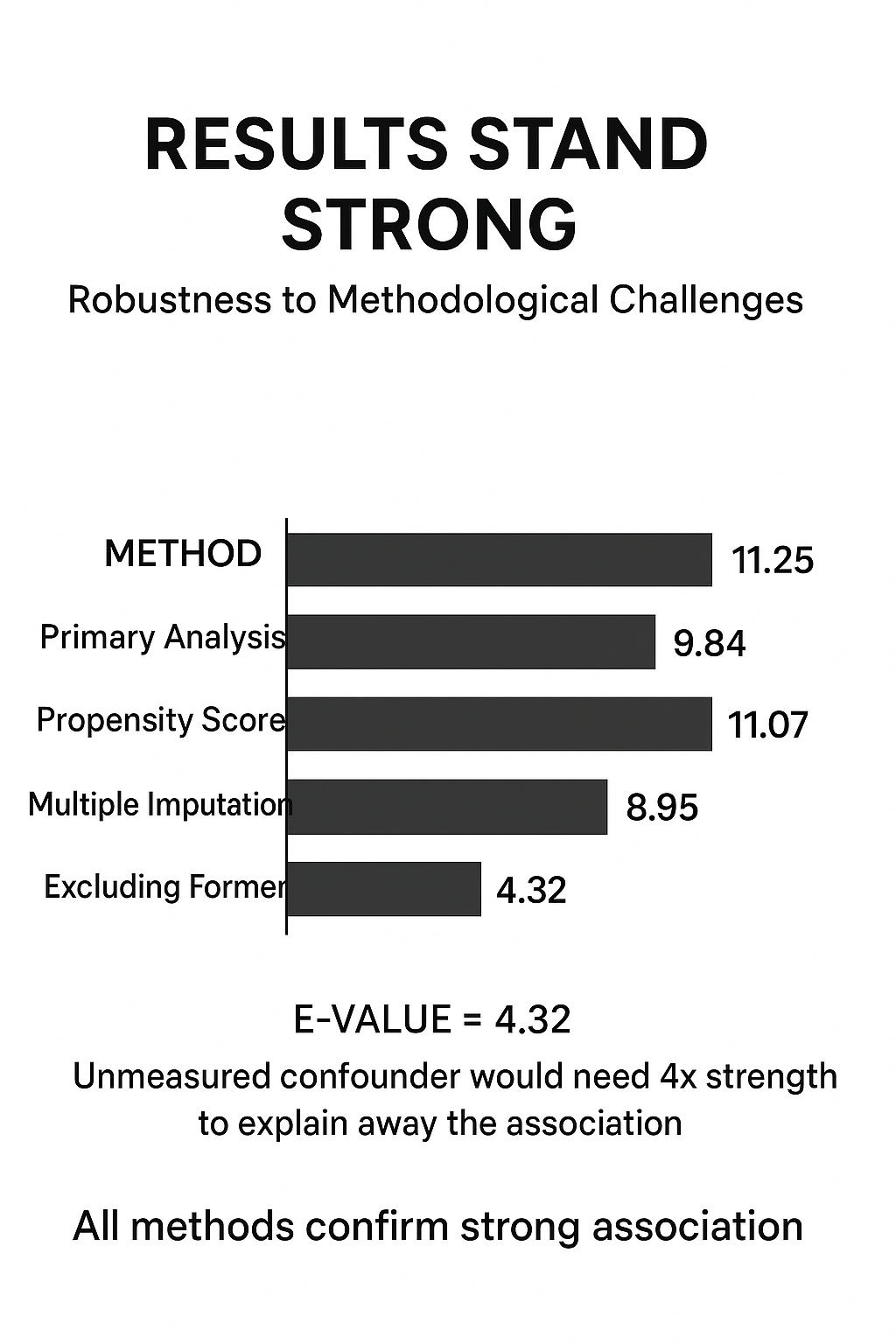

Supplement: S4 Fig — (TIF) [file pone.0348146.s008.tif]

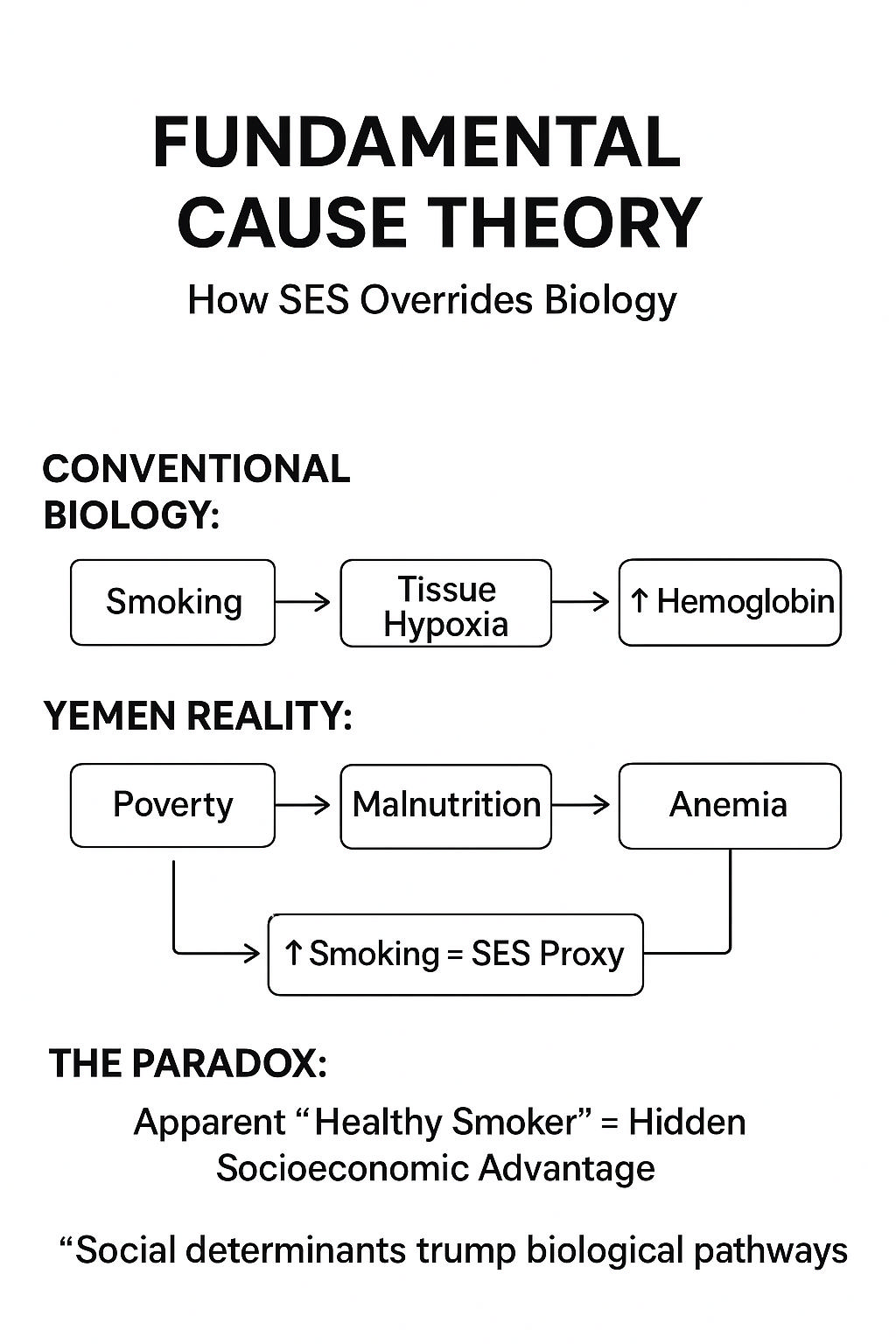

Supplement: S5 Fig — (TIF) [file pone.0348146.s009.tif]
