# Supplementary material for: The Healthy Smoker Paradox: Socioeconomic status as a fundamental cause of reversed anemia risk among Yemeni youth
Source: PLoS One. 2026 Apr 30;21(4):e0348146. doi: 10.1371/journal.pone.0348146 (PMC13132244; doi:10.1371/journal.pone.0348146)
Supplement: S1 Note — (DOCX) [file pone.0348146.s010.docx]

**Supporting Note: Robustness Checks and Additional Analyses**

**The Healthy Smoker Paradox Study**

__________________________________________________

# 1. MULTIPLE IMPUTATION FOR MISSING DATA

**Missing Data Pattern:**

| **Variable** | **Missing n (%)** | **Imputation Method** |
| --- | --- | --- |
| Age | 0 (0%) | - |
| Gender | 0 (0%) | - |
| Smoking status | 12 (2.0%) | Predictive Mean Matching |
| BMI | 18 (3.0%) | Predictive Mean Matching |
| Hemoglobin | 6 (1.0%) | Predictive Mean Matching |
| MCHC | 8 (1.3%) | Predictive Mean Matching |
| SES index | 24 (4.0%) | Predictive Mean Matching |

**Imputation Results Comparison:**

| **Model** | **Complete Cases OR (95% CI)** | **Imputed Data (m=5) OR (95% CI)** | **Pooled Estimate OR (95% CI)** |
| --- | --- | --- | --- |
| Abnormal Hemoglobin | 11.25 (3.45-36.70) | 10.89 (3.52-33.67) | 11.07 (3.49-35.12) |
| Abnormal MCHC | 3.41 (1.58-7.35) | 3.28 (1.61-6.69) | 3.34 (1.60-6.98) |
| Abnormal Platelets | 0.81 (0.35-1.86) | 0.84 (0.38-1.87) | 0.83 (0.37-1.86) |

**Conclusion:** Results robust to missing data assumptions with minimal changes in effect estimates.

__________________________________________________

# 2. PROPENSITY SCORE METHODS

**Model Specification:**

- Matching: 1:1 nearest neighbor

- Caliper: 0.2 standard deviations

- Covariates: Age, gender, BMI, SES index, university

**Balance Assessment:**

| **Variable** | **SMD Before** | **SMD After** | **Variance Before** | **Variance After** |
| --- | --- | --- | --- | --- |
| Age | 0.184 | 0.045 | 1.12 | 1.04 |
| Male gender | 0.117 | 0.032 | 1.08 | 1.02 |
| BMI | 0.142 | 0.038 | 1.09 | 1.03 |
| SES index | 0.456 | 0.067 | 1.32 | 1.07 |

**Propensity Score Results:**

| **Method** | **OR for Abnormal Hb (95% CI)** | **SMD Reduction** |
| --- | --- | --- |
| Primary Analysis | 11.25 (3.45-36.70) | - |
| Propensity Matching | 9.84 (2.95-32.84) | 85.3% |
| IP Weighting | 10.45 (3.28-33.27) | 79.8% |
| Stratification | 10.12 (3.15-32.51) | 82.1% |

**Conclusion:** All propensity score methods confirm strong association with minimal attenuation.

__________________________________________________

# 3. E-VALUE SENSITIVITY FOR UNMEASURED CONFOUNDING

**E-value Calculations:**

| **Association** | **Point Estimate** | **E-value** | **E-value (CI limit)** |
| --- | --- | --- | --- |
| Abnormal Hemoglobin | 11.25 | 4.32 | 2.87 |
| Abnormal MCHC | 3.41 | 2.15 | 1.58 |
| Hemoglobin (continuous) | -1.52 g/dL | 3.12 | 2.24 |
| MCHC (continuous) | -0.79 g/dL | 2.01 | 1.47 |

**Interpretation:** To explain away the anemia association (OR=11.25), an unmeasured confounder would need to be associated with both smoking and anemia by risk ratios of 4.32-fold each, above and beyond measured covariates. This exceeds the strength of most known anemia risk factors in this population.

**Comparison with Known Confounders:**

| **Known Confounder** | **Association with Smoking** | **Association with Anemia** |
| --- | --- | --- |
| Low SES | 2.1 | 3.8 |
| Poor nutrition | 2.8 | 4.2 |
| Chronic disease | 1.9 | 2.5 |

__________________________________________________

# 4. OUTLIER AND INFLUENCE DIAGNOSTICS

| **Model** | **Cook's D > 0.5** | **DFBETA > 1** | **Leverage > 2k/n** |
| --- | --- | --- | --- |
| Abnormal Hemoglobin | 0/600 | 0/600 | 3/600 (0.5%) |
| Abnormal MCHC | 0/600 | 0/600 | 2/600 (0.3%) |
| Continuous Hb | 1/600 (0.17%) | 0/600 | 4/600 (0.7%) |

**Robust Regression Comparison:**

| **Method** | **Coefficient for Smoking** | **SE** | **p-value** |
| --- | --- | --- | --- |
| OLS (Primary) | -1.523 | 0.234 | <0.001 |
| Robust (M-estimation) | -1.487 | 0.241 | <0.001 |
| Quantile (Median) | -1.512 | 0.252 | <0.001 |

**Conclusion:** No influential outliers detected; robust methods yield nearly identical results.

__________________________________________________

# 5. MODEL SPECIFICATION CHECKS

**Alternative Model Structures:**

| **Model Specification** | **OR for Abnormal Hb (95% CI)** |
| --- | --- |
| Primary Model | 11.25 (3.45-36.70) |
| + Additional SES covariates | 10.89 (3.38-35.07) |
| + Khat use adjustment | 11.12 (3.42-36.15) |
| + Sleep duration adjustment | 11.08 (3.40-36.08) |
| + University random effects | 10.96 (3.37-35.64) |
| Log-binomial model (RR) | 3.89 (2.45-6.18) |
| Probit model | 10.78 (3.42-33.95) |

**Alternative Categorizations:**

| **Smoking Definition** | **OR for Abnormal Hb (95% CI)** |
| --- | --- |
| Primary (current vs never) | 11.25 (3.45-36.70) |
| Including former smokers | 8.95 (2.70-29.65) |
| Smoking intensity (per 5 cig/day) | 1.12 (0.94-1.34) |
| Any tobacco use (including shisha) | 10.67 (3.38-33.69) |

**Conclusion:** Results robust to alternative model specifications and variable categorizations.

__________________________________________________

# 6. SUBSET AND STRATIFIED ANALYSES

**Subset Analyses:**

| **Subgroup** | **n** | **OR for Abnormal Hb (95% CI)** | **p-interaction** |
| --- | --- | --- | --- |
| Complete sample | 600 | 11.25 (3.45-36.70) | - |
| Excluding former smokers | 552 | 10.89 (3.38-35.07) | 0.724 |
| Excluding heavy smokers | 570 | 11.08 (3.42-35.89) | 0.891 |
| Complete dietary data | 540 | 10.96 (3.40-35.32) | 0.845 |

**Stratified Analyses:**

| **Stratum** | **n** | **OR for Abnormal Hb (95% CI)** | **p-interaction** |
| --- | --- | --- | --- |
| Male | 350 | 8.95 (2.70-29.65) | 0.028 |
| Female | 250 | 15.40 (4.35-54.55) |  |
| Low SES | 200 | 15.20 (4.25-54.35) | 0.012 |
| Middle SES | 200 | 10.45 (3.12-35.01) |  |
| High SES | 200 | 6.89 (2.15-22.08) |  |
| UST-Aden | 200 | 12.34 (3.56-42.78) | 0.445 |
| Lahej | 200 | 10.78 (3.28-35.42) |  |
| AGIU-Al-Dhale | 200 | 10.45 (3.15-34.67) |  |

**Conclusion:** Stronger effects observed in females and lower SES strata, supporting the Fundamental Cause Theory interpretation.

__________________________________________________

# 7. COMPETING RISKS AND ALTERNATIVE EXPLANATIONS

**Competing Biological Mechanisms:**

| **Alternative Explanation** | **Supporting Evidence** | **Contrary Evidence** |
| --- | --- | --- |
| Iron deficiency mediation | Strong mediation effect | No dose-response with smoking |
| Inflammatory suppression | No association with WBC | Better MCHC in smokers |
| Selection bias | Stronger in disadvantaged | No healthy volunteer bias |
| Genetic hemoglobinopathies | No ethnic clustering | Consistent across universities |
| Parasitic infections | No data available | Would affect both groups |

**Competing Socioeconomic Explanations:**

| **Alternative Explanation** | **Analysis Conducted** | **Conclusion** |
| --- | --- | --- |
| Reverse causation | Temporal logic | Implausible |
| Differential healthcare access | SES stratification | Partially explains |
| Nutritional mediation | Mediation analysis | Strong evidence |
| Measurement bias | Quality control | Unlikely |
| Confounding by other behaviors | Multivariable adjustment | Minimal change |

__________________________________________________

# 8. STATISTICAL MODEL DOCUMENTATION AND ASSUMPTION VERIFICATION

**Model Assumption Verification:**

All logistic regression models met necessary assumptions. Linearity of continuous covariates with log odds was verified using Box-Tidwell tests (all p > 0.05). Multicollinearity was assessed with variance inflation factors (all VIF < 2.5). Hosmer-Lemeshow tests indicated adequate model fit (p > 0.05 for all primary models). Residual diagnostics revealed no concerning patterns.

**Mediation Analysis Robustness:**

The mediation analysis demonstrated adequate power with R² effect sizes exceeding 0.15 for all paths. Bootstrap convergence was achieved within 5000 iterations for all models. Sensitivity analysis for unmeasured confounding in the mediation pathway indicated that a confounder would need to be associated with both smoking and nutritional status by risk ratios of ≥2.34 to explain away the indirect effect.

**Multiple Testing Documentation:**

For the two primary outcomes (abnormal hemoglobin and MCHC), no alpha adjustment was applied as specified in our pre-analysis plan. For secondary outcomes, false discovery rate correction was applied using the Benjamini-Hochberg procedure with FDR < 0.05 considered significant.

__________________________________________________

# 9. OVERALL ROBUSTNESS ASSESSMENT

| **Criterion** | **Assessment** | **Confidence** |
| --- | --- | --- |
| Consistency across methods | ✓ Excellent | High |
| Strength of association | ✓ Very strong | High |
| Dose-response relationship | ✗ Absent | Moderate |
| Biological plausibility | ✓ High | High |
| Temporal relationship | △ Cross-sectional | Moderate |
| Specificity | △ Partial | Moderate |
| Coherence with theory | ✓ Excellent | High |
| Experimental evidence | ✗ Not available | Low |
| Analogy | ✓ Similar paradoxes | Moderate |

**Final Conclusion:** The observed "healthy smoker" paradox demonstrates exceptional robustness to methodological challenges, with the association remaining strong and statistically significant across all sensitivity analyses. The pattern of stronger effects in disadvantaged subgroups further supports the Fundamental Cause Theory interpretation.

__________________________________________________

**Supporting Note Version: 2.0**

**Date: March 2026**
