# Supplementary material for: The Healthy Smoker Paradox: Socioeconomic status as a fundamental cause of reversed anemia risk among Yemeni youth
Source: PLoS One. 2026 Apr 30;21(4):e0348146. doi: 10.1371/journal.pone.0348146 (PMC13132244; doi:10.1371/journal.pone.0348146)
Supplement: S1 Methods — (DOCX) [file pone.0348146.s011.docx]

Supporting Methods: Statistical Power Considerations and Variable Coding Details

**The Healthy Smoker Paradox Study**

---

# 1. STATISTICAL POWER CONSIDERATIONS

## 1.1 Sample Size Calculation

An a priori power analysis was conducted using G*Power version 3.1.9.7. The following parameters were used:

- Alpha (α) = 0.05 (two-tailed)
- Power (1-β) = 0.80
- Anticipated effect size (odds ratio) = 2.5 (based on pilot data from the same population)
- Smoking prevalence = 24% (based on university student health statistics in Yemen)
- Anemia prevalence in non-smokers = 34% (based on pilot data)

The required sample size was calculated as 552 participants. Accounting for an anticipated 8% attrition or incomplete data, we targeted enrollment of 600 participants (200 per university).

## 1.2 Power for Subgroup Analyses

The sample size of 600 participants provided adequate power (≥80%) for the following subgroup analyses:

| **Subgroup** | **Sample Size** | **Detectable Effect Size (OR)** |
| --- | --- | --- |
| Gender (Male) | 350 | 2.8 |
| Gender (Female) | 250 | 3.2 |
| SES Tertile (Low) | 200 | 3.5 |
| SES Tertile (Medium) | 200 | 3.5 |
| SES Tertile (High) | 200 | 3.5 |
| University site | 200 each | 3.5 |

## 1.3 Power for Mediation Analysis

For mediation analysis using bootstrap methods with 5000 resamples, the sample size provided 80% power to detect moderate mediation effects (f² = 0.15) with bias-corrected confidence intervals.

## 1.4 Power for Interaction Testing

The sample size provided 80% power to detect interaction effects with odds ratios of 3.0 or greater for the primary exposure-confounder interaction terms.

---

# 2. VARIABLE CODING DETAILS

## 2.1 Socioeconomic Status (SES) Index

The SES index was constructed using principal component analysis (PCA) of the following 12 household assets and characteristics:

| **Component** | **Coding** |
| --- | --- |
| Parental education (father) | 1 = None, 2 = Primary, 3 = Secondary, 4 = University, 5 = Postgraduate |
| Parental education (mother) | Same as above |
| Radio ownership | 0 = No, 1 = Yes |
| Television ownership | 0 = No, 1 = Yes |
| Refrigerator ownership | 0 = No, 1 = Yes |
| Computer ownership | 0 = No, 1 = Yes |
| Smartphone ownership | 0 = No, 1 = Yes |
| Car ownership | 0 = No, 1 = Yes |
| Internet access at home | 0 = No, 1 = Yes |
| Water source | 1 = Piped, 2 = Well, 3 = Purchased |
| Sanitation type | 1 = Private toilet, 2 = Shared toilet |
| Food insecurity score | 0-12 (higher = more insecure) |

The first principal component (explaining 42.3% of variance) was extracted and standardized to create the SES index (mean = 0, SD = 1). Participants were then categorized into tertiles: Low SES (bottom 33.3%), Medium SES (middle 33.3%), and High SES (top 33.3%).

## 2.2 Smoking Status Classification

| **Category** | **Definition** |
| --- | --- |
| Never-smoker | Smoked less than 100 cigarettes in lifetime |
| Former smoker | Smoked 100 or more cigarettes in lifetime but currently not smoking |
| Current smoker | Smoked 100 or more cigarettes in lifetime AND currently smokes daily or occasionally |

## 2.3 Smoking Intensity Categories (Current Smokers Only)

| **Category** | **Cigarettes per day** |
| --- | --- |
| Light | 1-5 |
| Moderate | 6-10 |
| Heavy | 11-20 |
| Very Heavy | More than 20 |

## 2.4 BMI Categories (WHO Criteria)

| **Category** | **BMI (kg/m²)** |
| --- | --- |
| Underweight | < 18.5 |
| Normal | 18.5 - 24.9 |
| Overweight | 25 - 29.9 |
| Obese | ≥ 30 |

## 2.5 Hematological Abnormalities (Definitions)

| **Outcome** | **Definition** | **Source** |
| --- | --- | --- |
| Anemia | Hemoglobin < 13 g/dL (males), < 12 g/dL (females) | WHO criteria |
| Abnormal MCHC | Mean Corpuscular Hemoglobin Concentration < 32 g/dL | ICSH guidelines |
| Microcytosis | Mean Corpuscular Volume < 80 fL | ICSH guidelines |
| Thrombocytopenia | Platelet count < 150 × 10³/μL | ICSH guidelines |
| Abnormal PT | Prothrombin time > 14 seconds | Local reference range |
| Abnormal APTT | Activated Partial Thromboplastin Time > 38 seconds | Local reference range |

ICSH = International Council for Standardization in Haematology

## 2.6 Dietary Diversity Score

The dietary diversity score was calculated based on 24-hour recall of consumption from 9 food groups:

| **Food Group** | **Points** |
| --- | --- |
| Grains (bread, rice, pasta) | 1 |
| Vegetables | 1 |
| Fruits | 1 |
| Meat, poultry, or fish | 1 |
| Eggs | 1 |
| Milk or dairy products | 1 |
| Beans, lentils, or nuts | 1 |
| Fats or oils | 1 |
| Sweets or sugary drinks | 1 |

Total Score Range: 0 to 9 (higher score indicates greater dietary diversity)

## 2.7 Khat Chewing Frequency

| **Category** | **Definition** |
| --- | --- |
| Never | No khat chewing |
| Occasionally | 1-2 times per week |
| Weekly | 3-4 times per week |
| Daily | Daily consumption |

## 2.8 Sleep Duration Categories

| **Category** | **Hours per night** |
| --- | --- |
| Short sleep | < 7 hours |
| Normal sleep | 7-8 hours |
| Long sleep | > 8 hours |

---

# 3. STATISTICAL SOFTWARE AND PACKAGES

| **Software/Package** | **Version** | **Purpose** |
| --- | --- | --- |
| R | 4.3.1 | Primary statistical analysis |
| IBM SPSS Statistics | 28 | Data management and secondary analyses |
| mediation (R package) | 4.5.0 | Causal mediation analysis |
| MatchIt (R package) | 4.5.1 | Propensity score matching |
| EValue (R package) | 4.1.2 | Sensitivity analysis for unmeasured confounding |
| mice (R package) | 3.15.0 | Multiple imputation |
| car (R package) | 3.1-2 | Multicollinearity assessment |

---

# 4. MULTIPLE TESTING CORRECTION

For the two primary outcomes (abnormal hemoglobin and abnormal MCHC), no alpha adjustment was applied as specified in our pre-analysis plan. For secondary outcomes, false discovery rate (FDR) correction was applied using the Benjamini-Hochberg procedure with FDR < 0.05 considered statistically significant.

---

# 5. MISSING DATA HANDLING

Missing data patterns were assessed using Little's MCAR test (χ² = 15.23, p = 0.234), supporting the missing completely at random assumption. Primary analyses used complete cases given low missingness (< 5% for all primary variables). Sensitivity analyses with multiple imputation (m=5 datasets) using predictive mean matching produced nearly identical results.

---

*Supporting Methods Version: 2.0
Date: March 2026*
