# Supplementary material for: The Healthy Smoker Paradox: Socioeconomic status as a fundamental cause of reversed anemia risk among Yemeni youth
Source: PLoS One. 2026 Apr 30;21(4):e0348146. doi: 10.1371/journal.pone.0348146 (PMC13132244; doi:10.1371/journal.pone.0348146)
Supplement: S1 File — (DOCX) [file pone.0348146.s012.docx]

**Supporting Information 1**

**COMPREHENSIVE STUDY QUESTIONNAIRE - ENGLISH/ARABIC**

**=== SECTION A: BASIC DEMOGRAPHICS ===**

A1. Age: ______ years (العمر: ______ سنة)

A2. Gender: □ Male □ Female (الجنس: □ ذكر □ أنثى)

A3. Date of Birth: __/__/____ (تاريخ الميلاد: __/__/____)

A4. Place of Birth: ________________ (مكان الميلاد: ________________)

A5. Current Residence: □ Urban □ Rural (السكن الحالي: □ حضر □ ريف)

**=== SECTION B: ACADEMIC INFORMATION ===**

**B1. University:**

□ University of Science and Technology - Aden (جامعة العلوم والتكنولوجيا - عدن)

□ University of Lahej (جامعة لحج)

□ Aden Gulf International University - Al-Dhale (جامعة عدن الخليج الدولية - الضالع)

**B2. Faculty:**

□ Medical Sciences (العلوم الطبية)

□ Engineering (الهندسة)

□ Humanities (العلوم الإنسانية)

**B3. Academic Year:**

□ First Year (السنة الأولى)

□ Second Year (السنة الثانية)

□ Third Year (السنة الثالثة)

□ Fourth Year (السنة الرابعة)

**=== SECTION C: SOCIOECONOMIC STATUS ASSESSMENT ===**

**C1. PARENTAL EDUCATION (التعليم الأبوي):**

Father's Education:

□ No formal education (بدون تعليم)

□ Primary school (ابتدائي)

□ Secondary school (ثانوي)

□ University (جامعي)

□ Postgraduate (دراسات عليا)

Mother's Education:

□ No formal education (بدون تعليم)

□ Primary school (ابتدائي)

□ Secondary school (ثانوي)

□ University (جامعي)

□ Postgraduate (دراسات عليا)

**C2. HOUSEHOLD ASSETS (ممتلكات الأسرة):**

Please check all items your household owns:

□ Radio (راديو) □ Television (تلفاز) □ Refrigerator (ثلاجة)

□ Computer (كمبيوتر) □ Laptop (لابتوب) □ Car (سيارة)

□ Motorcycle (دراجة نارية) □ Smartphone (هاتف ذكي)

□ Internet access (إنترنت) □ Air conditioning (تكييف)

□ Washing machine (غسالة) □ Microwave (ميكروويف)

□ Water heater (سخان ماء) □ Solar panels (ألواح شمسية)

**C3. HOUSING CONDITIONS (ظروف السكن):**

Type of residence: □ Owned house (منزل مملوك) □ Rented house (منزل مؤجر)

Number of rooms: ______ (عدد الغرف: ______)

Number of people in household: ______ (عدد أفراد الأسرة: ______)

Water source: □ Piped water (مياه شبكة) □ Well (بئر) □ Purchased (مشترى)

Sanitation: □ Private toilet (مرحاض خاص) □ Shared toilet (مرحاض مشترك)

**C4. FOOD SECURITY (الأمن الغذائي):**

In the past 30 days, how often did you:

- Worry about not having enough food?

□ Never (أبداً) □ Rarely (نادراً) □ Sometimes (أحياناً) □ Often (كثيراً)

- Skip meals because there wasn't enough money for food?

□ Never (أبداً) □ Rarely (نادراً) □ Sometimes (أحياناً) □ Often (كثيراً)

- Eat less than you felt you should because of lack of money?

□ Never (أبداً) □ Rarely (نادراً) □ Sometimes (أحياناً) □ Often (كثيراً)

- Experience hunger but didn't eat because you couldn't afford food?

□ Never (أبداً) □ Rarely (نادراً) □ Sometimes (أحياناً) □ Often (كثيراً)

**=== SECTION D: SMOKING ASSESSMENT ===**

**D1. SMOKING STATUS (حالة التدخين):**

□ Never smoker - smoked less than 100 cigarettes in lifetime

(غير مدخن - دخن أقل من 100 سيجارة في العمر)

□ Former smoker - smoked ≥100 cigarettes but quit

(مدخن سابق - دخن 100 سيجارة أو أكثر ولكن أقلع)

□ Current smoker - smoked ≥100 cigarettes and currently smokes

(مدخن حالي - دخن 100 سيجارة أو أكثر ويدخن حالياً)

**D2. SMOKING HISTORY (FOR CURRENT SMOKERS) (تاريخ التدخين للمدخنين الحاليين):**

Age when you started smoking regularly: ______ years

(العمر عند بداية التدخين المنتظم: ______ سنة)

How many years have you been smoking? ______ years

(كم سنة وأنت تدخن؟ ______ سنة)

Number of cigarettes per day:

□ 1-5 □ 6-10 □ 11-20 □ More than 20 (أكثر من 20)

(عدد السجائر يومياً: □ 1-5 □ 6-10 □ 11-20 □ أكثر من 20)

**D3. TYPE OF TOBACCO PRODUCTS (أنواع منتجات التبغ):**

Which tobacco products do you currently use? (Check all that apply)

(أي منتجات التبغ تستخدمها حالياً؟ (اختر كل ما ينطبق))

□ Factory-made cigarettes (سجائر مصنعة)

□ Hand-rolled cigarettes (سجائر ملفوفة)

□ Shisha/Waterpipe (أرجيلة/شيشة)

□ Medwakh (مدوخ)

□ Other: ________________ (غير ذلك: ________________)

**D4. SECONDHAND SMOKE EXPOSURE (التعرض للتدخين السلبي**):

Does anyone smoke inside your home?

(هل يدخن أي شخص داخل منزلك؟)

□ No (لا) □ Yes, occasionally (نعم، أحياناً) □ Yes, daily (نعم، يومياً)

**=== SECTION E: NUTRITIONAL ASSESSMENT ===**

**E1. DIETARY DIVERSITY (تنوع الغذاء):**

Yesterday, did you eat or drink any of the following? (Check all that apply)

(أمس، هل أكلت أو شربت أي من التالي؟ (اختر كل ما ينطبق))

□ Grains (bread, rice, pasta, etc.) (الحبوب: خبز، أرز، معكرونة، إلخ)

□ Vegetables (الخضروات)

□ Fruits (الفواكه)

□ Meat, poultry, or fish (اللحوم، الدواجن، أو الأسماك)

□ Eggs (البيض)

□ Milk or dairy products (الحليب أو منتجات الألبان)

□ Beans, lentils, or nuts (الفول، العدس، أو المكسرات)

□ Fats or oils (الدهون أو الزيوت)

□ Sweets or sugary drinks (الحلويات أو المشروبات السكرية)

**E2. MEAL PATTERNS (أنماط الوجبات):**

How many meals do you typically eat per day? ______ meals

(كم وجبة تأكل عادة في اليوم؟ ______ وجبات)

Do you usually eat breakfast? □ Yes □ No

(هل تتناول الفطور عادة؟ □ نعم □ لا)

**E3. NUTRITIONAL SUPPLEMENTATION (المكملات الغذائية):**

Are you currently taking any of the following? (Check all that apply)

(هل تتناول حالياً أي من التالي؟ (اختر كل ما ينطبق))

□ Iron supplements (مكملات الحديد)

□ Vitamin supplements (مكملات الفيتامينات)

□ Other nutritional supplements (مكملات غذائية أخرى)

□ None (لا شيء)

**=== SECTION F: OTHER HEALTH BEHAVIORS ===**

**F1. KHAT CHEWING (تناول القات):**

How often do you chew khat?

(كم مرة تمضغ القات؟)

□ Never (أبداً)

□ Occasionally (1-2 times per week) (أحياناً (1-2 مرة في الأسبوع))

□ Weekly (3-4 times per week) (أسبوعياً (3-4 مرات في الأسبوع))

□ Daily (يومياً)

**F2. SLEEP PATTERNS (أنماط النوم):**

How many hours do you usually sleep per night? ______ hours

(كم ساعة تنام عادة في الليل؟ ______ ساعات)

Sleep quality: □ Very good (ممتاز) □ Good (جيد) □ Fair (متوسط) □ Poor (سيء)

(جودة النوم: □ ممتاز □ جيد □ متوسط □ سيء)

**F3. PHYSICAL ACTIVITY (النشاط البدني):**

How many days per week do you engage in moderate to vigorous physical activity?

(كم يوم في الأسبوع تمارس نشاط بدني متوسط إلى شديد؟)

□ None (لا شيء) □ 1-2 days □ 3-4 days □ 5 or more days (5 أيام أو أكثر)

**=== SECTION G: MEDICAL HISTORY ===**

**G1. CURRENT HEALTH CONDITIONS (الحالات الصحية الحالية):**

Have you been diagnosed with any of the following? (Check all that apply)

(هل تم تشخيصك بأي من الحالات التالية؟ (اختر كل ما ينطبق))

□ Anemia (فقر الدم)

□ Iron deficiency (نقص الحديد)

□ Other nutritional deficiencies (نقص تغذوي آخر)

□ Chronic diseases (أمراض مزمنة)

□ None of the above (لا شيء مما سبق)

**G2. MEDICATION USE (استخدام الأدوية):**

Are you currently taking any medications regularly? □ Yes □ No

(هل تتناول أي أدوية بانتظام حالياً؟ □ نعم □ لا)

If yes, please specify: ________________ (إذا نعم، يرجى التحديد: ________________)

**=== INFORMED CONSENT STATEMENT ===**

"I have read and understood the information about this study. I have had the opportunity to ask questions and have received satisfactory answers. I voluntarily agree to participate in this study and provide blood samples for hematological analysis. I understand that my information will be kept confidential and used only for research purposes. I understand that I may withdraw from the study at any time without giving a reason."

("لقد قرأت وفهمت المعلومات حول هذه الدراسة. وقد أتيحت لي الفرصة لطرح الأسئلة وتلقيت إجابات مرضية. أوافق طواعية على المشاركة في هذه الدراسة وتقديم عينات الدم للتحليل الدموي. وأفهم أن معلوماتي سيتم الحفاظ على سريتها واستخدامها لأغراض البحث فقط. وأفهم أنه يمكنني الانسحاب من الدراسة في أي وقت دون إبداء أسباب.")

Participant Signature: _________________________ Date: _________________________

(توقيع المشارك: _________________________ التاريخ: _________________________)

Researcher Signature: _________________________ Date: _________________________

(توقيع الباحث: _________________________ التاريخ: _________________________)
