# Supplementary material for: The Healthy Smoker Paradox: Socioeconomic status as a fundamental cause of reversed anemia risk among Yemeni youth
Source: PLoS One. 2026 Apr 30;21(4):e0348146. doi: 10.1371/journal.pone.0348146 (PMC13132244; doi:10.1371/journal.pone.0348146)
Supplement: S2 File — (DOCX) [file pone.0348146.s013.docx]

**Supporting Information 2**

**COMPREHENSIVE LABORATORY PROTOCOL AND QUALITY ASSURANCE**

**1.0 SAMPLE COLLECTION PROTOCOL**

**1.1 Pre-collection Preparation**

- Verify participant fasting status (8-12 hours overnight fast)

- Confirm completion of informed consent form

- Check participant identification against study records

- Prepare necessary supplies:

Tourniquet

70% alcohol swabs

21G sterile needles

K3 EDTA tubes (lavender top) - 2 mL

Sodium citrate tubes (blue top) - 2 mL

Tube holders and safety devices

Bandages and gauze

Biohazard sharps container

Sample transport cooler

**1.2 Venipuncture Procedure**

1. Position participant comfortably in sitting position

2. Apply tourniquet 3-4 inches above intended puncture site

3. Select appropriate vein (median cubital vein preferred)

4. Disinfect site with 70% alcohol using circular motion

5. Allow site to air dry completely (30 seconds)

6. Perform venipuncture using 21G needle

7. Collect blood in following order:

- First: K3 EDTA tube (2 mL)

- Second: Sodium citrate tube (2 mL)

8. Release tourniquet immediately after blood flow established

9. Apply gentle pressure with gauze after needle withdrawal

10. Label tubes immediately with participant ID and time

**1.3 Sample Processing**

- EDTA tubes: Mix by gentle inversion 8-10 times immediately after collection

- Citrate tubes: Ensure exact fill volume, mix by gentle inversion 3-4 times

- Store samples at room temperature (18-25°C)

- Process all samples within 4 hours of collection

- Document any deviations from protocol

**2.0 ANALYTICAL METHODS**

**2.1 Hematology Analysis (Mindray BC-3000 Plus)**

**Instrument Settings:**

- Analysis mode: Standard CBC mode

- Aspiration volume: 20 μL

- Dilution ratio: 1:224

- Counting time: 10 seconds

**Parameters Measured:**

- White Blood Cell Count (WBC)

- Red Blood Cell Count (RBC)

- Hemoglobin (Hb)

- Hematocrit (HCT)

- Mean Corpuscular Volume (MCV)

- Mean Corpuscular Hemoglobin (MCH)

- Mean Corpuscular Hemoglobin Concentration (MCHC)

- Platelet Count (PLT)

- Red Cell Distribution Width (RDW)

**Analytical Principle:**

- WBC: Impedance method with differential lysis

- RBC/PLT: Impedance method

- Hb: Cyanide-free photometric method

**2.2 Coagulation Analysis (BA-88A Semi-automated)**

**Prothrombin Time (PT) Protocol:**

1. Pre-warm reagents and samples to 37°C

2. Mix 50 μL platelet-poor plasma with 100 μL PT reagent

3. Incubate exactly 3 minutes at 37°C

4. Add 50 μL 0.025 M calcium chloride

5. Measure clotting time automatically

**Activated Partial Thromboplastin Time (APTT) Protocol:**

1. Mix 50 μL platelet-poor plasma with 50 μL APTT reagent

2. Incubate exactly 5 minutes at 37°C

3. Add 50 μL 0.025 M calcium chloride

4. Measure clotting time automatically

**3.0 QUALITY ASSURANCE PROGRAM**

**3.1 Internal Quality Control**

**Daily Procedures:**

- Run three-level commercial controls (low, normal, high) with each batch

- Document all QC results in laboratory logbook

- Calculate means and standard deviations monthly

- Plot results on Levey-Jennings charts

**Acceptance Criteria:**

- Hemoglobin: CV ≤ 3.0%

- MCHC: CV ≤ 2.0%

- Platelets: CV ≤ 5.0%

- PT/APTT: CV ≤ 5.0%

**Corrective Actions:**

- If one control level fails: Repeat analysis

- If two control levels fail: Check calibration and reagents

- If persistent failures: Instrument maintenance and troubleshooting

**3.2 External Quality Assessment**

**Program Participation:**

- International quality assurance program (Bio-Rad)

- Quarterly proficiency testing

- Independent evaluation of performance

**Performance Standards:**

- All parameters within ±2SD of peer group mean

- Timely submission of results

- Documentation of corrective actions for any failures

**3.3 Calibration Protocol**

**Frequency**:

- Full calibration: Every 6 months

- Partial calibration: Monthly

- Daily verification: Using system checks

**Calibration Materials:**

- Manufacturer-provided calibrators

- Traceable to international standards

- Documented certificate of analysis

**4.0 SAMPLE INTEGRITY AND STORAGE**

**4.1 Acceptance Criteria**

- No visible hemolysis

- No clots in EDTA samples

- Adequate fill volume (≥90% of stated volume)

- Proper labeling and identification

- Timely processing (<4 hours)

**4.2 Storage Conditions**

- Short-term: Room temperature (18-25°C) for ≤4 hours

- Medium-term: Refrigerated (2-8°C) for ≤24 hours

- Long-term: Frozen at -80°C for future studies

**4.3 Sample Rejection Criteria**

- Gross hemolysis

- Insufficient volume

- Incorrect anticoagulant

- Expired collection tubes

- Improper labeling

- Excessive time delay in processing

**5.0 SAFETY PROCEDURES**

**5.1 Universal Precautions**

- Wear appropriate PPE (gloves, lab coat, eye protection)

- No eating, drinking, or smoking in laboratory

- Proper hand hygiene before and after procedures

- Safe disposal of sharps and biohazard waste

**5.2 Emergency Procedures**

- Exposure to blood: Immediate washing and medical evaluation

- Spill cleanup: Using appropriate disinfectants

- Equipment malfunction: Immediate shutdown and reporting

**6.0 DOCUMENTATION AND RECORD KEEPING**

**6.1 Required Records**

- Sample collection log

- Instrument maintenance records

- Quality control results

- Calibration certificates

- Proficiency testing reports

- Incident reports

**6.2 Retention Period**

- All records maintained for minimum of 5 years

- Electronic backup of all data

- Secure storage of hard copies

**PROTOCOL VERSION: 2.0**

**EFFECTIVE DATE:** January 1, 2025

**APPROVED BY:** Laboratory Director
