# Supplementary material for: The Healthy Smoker Paradox: Socioeconomic status as a fundamental cause of reversed anemia risk among Yemeni youth
Source: PLoS One. 2026 Apr 30;21(4):e0348146. doi: 10.1371/journal.pone.0348146 (PMC13132244; doi:10.1371/journal.pone.0348146)
Supplement: S3 File — (DOCX) [file pone.0348146.s014.docx]

File S3: R Analysis Script: The Healthy Smoker Paradox Study

###############################################################################
# COMPREHENSIVE R ANALYSIS SCRIPT
# The Healthy Smoker Paradox Study
# Yemeni University Students (N=600)
###############################################################################

# =============================================================================
# 1.0 SETUP AND PACKAGE INSTALLATION
# =============================================================================

# Install required packages if not already installed
required_packages <- c("tidyverse", "ggplot2", "mediation", "MatchIt",
 "EValue", "mice", "car", "lme4", "boot")

for (pkg in required_packages) {
 if (!require(pkg, character.only = TRUE)) {
 install.packages(pkg)
 library(pkg, character.only = TRUE)
 }
}

# Set working directory (USER MUST UPDATE THIS PATH)
# setwd("path/to/your/data/folder")

# =============================================================================
# 2.0 DATA IMPORT AND PREPARATION
# =============================================================================

# Read the main dataset
df <- read.csv("File_S1_Raw_Data_Anonymized.csv")

# Check data structure
str(df)
summary(df)

# Convert categorical variables to factors
df$gender <- factor(df$gender, levels = c("Male", "Female"))
df$smoking <- factor(df$smoking, levels = c("Smoker", "Non-smoker"))
df$khat <- factor(df$khat, levels = c("Never", "Occasionally", "Weekly", "Daily"))
df$sleep_cat <- factor(df$sleep_cat, levels = c("<3h", "4-7h", "8-11h"))

# Create derived variables
df$anemia <- factor(df$hb_abn, levels = c(0, 1), labels = c("Normal", "Anemia"))
df$mchc_abn <- factor(df$mchc_abn, levels = c(0, 1), labels = c("Normal", "Abnormal"))

# Calculate nutritional composite score (if not already present)
df$nutrition_score <- scale(df$bmi + df$dietary_diversity + (10 - df$food_insecurity))

# =============================================================================
# 3.0 DESCRIPTIVE STATISTICS
# =============================================================================

# Table 1: Baseline characteristics by smoking status
library(table1)

table1_data <- df
table1_data$smoking <- factor(table1_data$smoking, labels = c("Smokers", "Non-Smokers"))

table1_output <- table1(~ age + gender + bmi + khat + sleep_cat + hb + mchc + plt + pt + ptt | smoking,
 data = table1_data)

print(table1_output)

# =============================================================================
# 4.0 PRIMARY ANALYSIS: MULTIVARIATE LOGISTIC REGRESSION
# =============================================================================

# Model 1: Abnormal Hemoglobin
model_hb_unadj <- glm(anemia ~ smoking, data = df, family = binomial)
model_hb_adj <- glm(anemia ~ smoking + age + gender + bmi + university,
 data = df, family = binomial)

summary(model_hb_adj)
exp(coef(model_hb_adj))
exp(confint(model_hb_adj))

# Model 2: Abnormal MCHC
model_mchc_unadj <- glm(mchc_abn ~ smoking, data = df, family = binomial)
model_mchc_adj <- glm(mchc_abn ~ smoking + age + gender + bmi + university,
 data = df, family = binomial)

summary(model_mchc_adj)
exp(coef(model_mchc_adj))
exp(confint(model_mchc_adj))

# =============================================================================
# 5.0 MEDIATION ANALYSIS
# =============================================================================

library(mediation)

# Model for mediator (nutritional status)
model_mediator <- lm(nutrition_score ~ smoking + age + gender + bmi + university,
 data = df)

# Model for outcome (hemoglobin)
model_outcome <- lm(hb ~ smoking + nutrition_score + age + gender + bmi + university,
 data = df)

# Mediation analysis with bootstrapping
mediation_results <- mediate(model_mediator, model_outcome,
 treat = "smoking", mediator = "nutrition_score",
 boot = TRUE, sims = 5000)

summary(mediation_results)

# =============================================================================
# 6.0 PROPENSITY SCORE MATCHING
# =============================================================================

library(MatchIt)

# Create propensity score model
ps_model <- matchit(smoking ~ age + gender + bmi + university,
 data = df, method = "nearest", distance = "logit",
 caliper = 0.2, ratio = 1)

# Extract matched data
matched_data <- match.data(ps_model)

# Balance assessment
summary(ps_model)
plot(ps_model, type = "jitter")

# Logistic regression on matched data
model_matched <- glm(anemia ~ smoking + age + gender + bmi + university,
 data = matched_data, family = binomial)

exp(coef(model_matched))
exp(confint(model_matched))

# =============================================================================
# 7.0 E-VALUE SENSITIVITY ANALYSIS
# =============================================================================

library(EValue)

# Calculate E-value for primary association
or_hb <- exp(coef(model_hb_adj)["smokingNon-smoker"])
ci_hb <- exp(confint(model_hb_adj))["smokingNon-smoker", ]

evalue_hb <- evalues.OR(or_hb, ci_hb[1], ci_hb[2], rare = FALSE)
print(evalue_hb)

# Calculate E-value for MCHC association
or_mchc <- exp(coef(model_mchc_adj)["smokingNon-smoker"])
ci_mchc <- exp(confint(model_mchc_adj))["smokingNon-smoker", ]

evalue_mchc <- evalues.OR(or_mchc, ci_mchc[1], ci_mchc[2], rare = FALSE)
print(evalue_mchc)

# =============================================================================
# 8.0 EFFECT MODIFICATION ANALYSES
# =============================================================================

# Interaction with Gender
model_gender_int <- glm(anemia ~ smoking * gender + age + bmi + university,
 data = df, family = binomial)

# Interaction with SES
model_ses_int <- glm(anemia ~ smoking * ses_tertile + age + gender + bmi + university,
 data = df, family = binomial)

# Likelihood ratio tests
anova(model_hb_adj, model_gender_int, test = "LRT")
anova(model_hb_adj, model_ses_int, test = "LRT")

# Stratified analyses
df_male <- subset(df, gender == "Male")
df_female <- subset(df, gender == "Female")
df_low_ses <- subset(df, ses_tertile == "Low")
df_high_ses <- subset(df, ses_tertile == "High")

model_male <- glm(anemia ~ smoking + age + bmi + university,
 data = df_male, family = binomial)
model_female <- glm(anemia ~ smoking + age + bmi + university,
 data = df_female, family = binomial)
model_low_ses <- glm(anemia ~ smoking + age + gender + bmi + university,
 data = df_low_ses, family = binomial)
model_high_ses <- glm(anemia ~ smoking + age + gender + bmi + university,
 data = df_high_ses, family = binomial)

# =============================================================================
# 9.0 SENSITIVITY ANALYSIS: MULTIPLE IMPUTATION
# =============================================================================

library(mice)

# Check missing data pattern
md_pattern <- md.pattern(df)

# Multiple imputation
imp <- mice(df, m = 5, method = "pmm", seed = 12345)

# Pooled logistic regression
pooled_hb <- with(imp, glm(anemia ~ smoking + age + gender + bmi + university,
 family = binomial))
summary(pool(pooled_hb))

# =============================================================================
# 10.0 SENSITIVITY ANALYSIS: EXCLUDING FORMER SMOKERS
# =============================================================================

# If former smokers are identified, exclude them
df_never_only <- subset(df, smoking_status != "Former")

model_never <- glm(anemia ~ smoking + age + gender + bmi + university,
 data = df_never_only, family = binomial)

exp(coef(model_never))
exp(confint(model_never))

# =============================================================================
# 11.0 VISUALIZATION
# =============================================================================

library(ggplot2)

# Figure 1: Prevalence of hemoglobin abnormalities
fig1_data <- df %>%
 group_by(smoking) %>%
 summarise(prev = mean(anemia == "Anemia") * 100,
 se = sqrt(prev * (100 - prev) / n()))

fig1 <- ggplot(fig1_data, aes(x = smoking, y = prev, fill = smoking)) +
 geom_bar(stat = "identity", width = 0.7) +
 geom_errorbar(aes(ymin = prev - 1.96 * se, ymax = prev + 1.96 * se),
 width = 0.2) +
 labs(title = "The Healthy Smoker Paradox",
 x = "Smoking Status", y = "Prevalence of Anemia (%)") +
 theme_minimal() +
 scale_fill_manual(values = c("#E41A1C", "#377EB8")) +
 theme(legend.position = "none")

ggsave("Figure1.png", fig1, width = 6, height = 4, dpi = 300)

# Figure 2: Mediation pathway
# Visualization of mediation results
mediation_plot_data <- data.frame(
 Effect = c("Total Effect", "Direct Effect", "Indirect Effect"),
 Estimate = c(-1.523, -0.945, -0.578),
 CI_lower = c(-1.982, -1.334, -0.867),
 CI_upper = c(-1.064, -0.556, -0.301)
)

fig2 <- ggplot(mediation_plot_data, aes(x = Effect, y = Estimate)) +
 geom_point(size = 3) +
 geom_errorbar(aes(ymin = CI_lower, ymax = CI_upper), width = 0.2) +
 geom_hline(yintercept = 0, linetype = "dashed", color = "red") +
 labs(title = "Mediation Through Nutritional Pathways",
 x = "Effect Type", y = "Effect Estimate (β)") +
 theme_minimal()

ggsave("Figure2.png", fig2, width = 6, height = 4, dpi = 300)

# =============================================================================
# 12.0 SUPPLEMENTARY ANALYSES
# =============================================================================

# Dose-response analysis
df_smokers <- subset(df, smoking == "Smoker")
df_smokers$intensity <- factor(df_smokers$cigarettes_per_day,
 levels = c("Light", "Moderate", "Heavy", "Very Heavy"))

model_dose <- glm(anemia ~ intensity + age + gender + bmi,
 data = df_smokers, family = binomial)

# Quantile regression
library(quantreg)

model_quantile <- rq(hb ~ smoking + age + gender + bmi + university,
 data = df, tau = c(0.25, 0.5, 0.75))
summary(model_quantile)

# Robust regression
library(MASS)

model_robust <- rlm(hb ~ smoking + age + gender + bmi + university,
 data = df)
summary(model_robust)

# =============================================================================
# 13.0 OUTPUT RESULTS TABLES
# =============================================================================

# Create summary table for primary results
results_table <- data.frame(
 Outcome = c("Abnormal Hemoglobin", "Abnormal MCHC"),
 OR = c(exp(coef(model_hb_adj)["smokingNon-smoker"]),
 exp(coef(model_mchc_adj)["smokingNon-smoker"])),
 CI_Lower = c(exp(confint(model_hb_adj))["smokingNon-smoker", 1],
 exp(confint(model_mchc_adj))["smokingNon-smoker", 1]),
 CI_Upper = c(exp(confint(model_hb_adj))["smokingNon-smoker", 2],
 exp(confint(model_mchc_adj))["smokingNon-smoker", 2]),
 P_Value = c(coef(summary(model_hb_adj))["smokingNon-smoker", 4],
 coef(summary(model_mchc_adj))["smokingNon-smoker", 4])
)

print(results_table)

# Save results to CSV
write.csv(results_table, "Primary_Results.csv", row.names = FALSE)

# =============================================================================
# 14.0 SESSION INFORMATION
# =============================================================================

sessionInfo()

# END OF SCRIPT
