# Supplementary material for: The Healthy Smoker Paradox: Socioeconomic status as a fundamental cause of reversed anemia risk among Yemeni youth
Source: PLoS One. 2026 Apr 30;21(4):e0348146. doi: 10.1371/journal.pone.0348146 (PMC13132244; doi:10.1371/journal.pone.0348146)
Supplement: S4 File — (DOCX) [file pone.0348146.s015.docx]

# Supporting Information 4 COMPREHENSIVE DATA DICTIONARY

| VARIABLE NAME | TYPE | DESCRIPTION | CODING/UNITS |
| --- | --- | --- | --- |
| id | Numeric | Unique participant identifier | Sequential 1-600 |
| age | Numeric | Participant age in years | Continuous, 18-25 |
| gender | Categorical | Participant gender | 1 = Male, 2 = Female |
| university | Categorical | University affiliation | 1 = UST-Aden, 2 = Lahej, 3 = AGIU |
| faculty | Categorical | Academic faculty | 1 = Medical, 2 = Engineering, 3 = Humanities |

## SOCIOECONOMIC VARIABLES

| VARIABLE NAME | TYPE | DESCRIPTION | CODING/UNITS |
| --- | --- | --- | --- |
| parent_edu_father | Categorical | Father's education level | 1 = None, 2 = Primary, 3 = Secondary, 4 = University, 5 = Postgraduate |
| parent_edu_mother | Categorical | Mother's education level | Same as above |
| household_assets | Numeric | Number of assets owned (0-15) | Continuous |
| has_car | Binary | Household car ownership | 0 = No, 1 = Yes |
| has_internet | Binary | Internet access at home | 0 = No, 1 = Yes |
| food_security | Numeric | Food security score (0-12) | Higher = more insecure |
| ses_index | Numeric | Composite SES index | Continuous, standardized |
| ses_tertile | Categorical | SES tertile | 1 = Low, 2 = Medium, 3 = High |

## SMOKING VARIABLES

| VARIABLE NAME | TYPE | DESCRIPTION | CODING/UNITS |
| --- | --- | --- | --- |
| smoking_status | Categorical | Current smoking status | 1 = Never, 2 = Former, 3 = Current |
| smoker | Binary | Current smoker vs never-smoker | 0 = Never, 1 = Current |
| cigarettes_per_day | Categorical | Daily cigarette consumption | 1 = 1-5, 2 = 6-10, 3 = 11-20, 4 = >20 |
| smoking_duration | Numeric | Years of regular smoking | Continuous |
| age_start_smoking | Numeric | Age started smoking | Continuous |
| tobacco_types | Categorical | Types of tobacco used | Multiple response allowed |
| secondhand_smoke | Categorical | Household secondhand exposure | 1 = None, 2 = Occasional, 3 = Daily |

## NUTRITIONAL VARIABLES

| VARIABLE NAME | TYPE | DESCRIPTION | CODING/UNITS |
| --- | --- | --- | --- |
| bmi | Numeric | Body Mass Index | kg/m² |
| bmi_category | Categorical | BMI classification | 1 = Underweight, 2 = Normal, 3 = Overweight, 4 = Obese |
| dietary_diversity | Numeric | Dietary diversity score (0-9) | Continuous |
| food_groups | Numeric | Number of food groups consumed | Continuous |
| breakfast_regular | Binary | Regular breakfast consumption | 0 = No, 1 = Yes |
| supplement_use | Binary | Current supplement use | 0 = No, 1 = Yes |
| nutritional_index | Numeric | Composite nutritional index | Continuous, standardized |

## OTHER BEHAVIORAL VARIABLES

| VARIABLE NAME | TYPE | DESCRIPTION | CODING/UNITS |
| --- | --- | --- | --- |
| khat_frequency | Categorical | Khat chewing frequency | 1 = Never, 2 = Occasionally, 3 = Weekly, 4 = Daily |
| khat_weekly | Binary | Weekly or daily khat use | 0 = No, 1 = Yes |
| sleep_duration | Categorical | Usual sleep duration | 1 = <7 hours, 2 = 7-8 hours, 3 = >8 hours |
| sleep_short | Binary | Short sleep duration | 0 = No, 1 = Yes |
| sleep_long | Binary | Long sleep duration | 0 = No, 1 = Yes |
| physical_activity | Categorical | Days of physical activity/week | 1 = None, 2 = 1-2, 3 = 3-4, 4 = ≥5 |

## HEMATOLOGICAL VARIABLES - CONTINUOUS

| VARIABLE NAME | TYPE | DESCRIPTION | CODING/UNITS |
| --- | --- | --- | --- |
| hemoglobin | Numeric | Hemoglobin concentration | g/dL |
| mchc | Numeric | Mean Corpuscular Hemoglobin Conc. | g/dL |
| mcv | Numeric | Mean Corpuscular Volume | fL |
| mch | Numeric | Mean Corpuscular Hemoglobin | pg |
| platelets | Numeric | Platelet count | ×10³/μL |
| wbc | Numeric | White blood cell count | ×10³/μL |
| rbc | Numeric | Red blood cell count | ×10⁶/μL |
| pt | Numeric | Prothrombin time | seconds |
| aptt | Numeric | Activated Partial Thromboplastin Time | seconds |

## HEMATOLOGICAL VARIABLES - CATEGORICAL

| VARIABLE NAME | TYPE | DESCRIPTION | CODING/UNITS |
| --- | --- | --- | --- |
| anemia | Binary | Presence of anemia | 0 = No, 1 = Yes |
| abnormal_mchc | Binary | Abnormal MCHC | 0 = No, 1 = Yes |
| abnormal_plt | Binary | Thrombocytopenia | 0 = No, 1 = Yes |
| microcytosis | Binary | Microcytosis (MCV <80 fL) | 0 = No, 1 = Yes |
| abnormal_pt | Binary | Prolonged PT | 0 = No, 1 = Yes |
| abnormal_aptt | Binary | Prolonged APTT | 0 = No, 1 = Yes |

## DERIVED VARIABLES

| VARIABLE NAME | TYPE | DESCRIPTION | CODING/UNITS |
| --- | --- | --- | --- |
| nutrition_mediator | Numeric | Mediation variable for nutrition | Continuous |
| pscore | Numeric | Propensity score | 0-1 |
| weights_psm | Numeric | Propensity score weights | Continuous |
| residuals_hb | Numeric | Regression residuals for Hb | Continuous |

## QUALITY CONTROL VARIABLES

| VARIABLE NAME | TYPE | DESCRIPTION | CODING/UNITS |
| --- | --- | --- | --- |
| sample_quality | Categorical | Blood sample quality | 1 = Excellent, 2 = Good, 3 = Acceptable, 4 = Poor |
| processing_time | Numeric | Time from collection to processing | Minutes |
| missing_data | Binary | Major data missing | 0 = No, 1 = Yes |
| outlier_flag | Binary | Statistical outlier | 0 = No, 1 = Yes |

**DEFINITION OF KEY OUTCOMES:**

- Anemia: Hemoglobin <13 g/dL for men, <12 g/dL for women (WHO criteria)

- Abnormal MCHC: <32 g/dL (International Council for Standardization in Haematology)

- Thrombocytopenia: Platelets <150 ×10³/μL

- Microcytosis: MCV <80 fL

- Prolonged PT: >14 seconds

- Prolonged APTT: >38 seconds

**DATA COLLECTION METHODS:**

- Hematological parameters: Automated analyzers with quality control

- Behavioral data: Structured questionnaires with trained interviewers

- Anthropometric measurements: Standard protocols with calibrated equipment

- Socioeconomic data: Validated instruments adapted for local context

**QUALITY ASSURANCE:**

- Double data entry with verification

- Range checks and logical consistency checks

- Random audit of 10% questionnaires

- Laboratory quality control participation
