# Supplementary material for: The Healthy Smoker Paradox: Socioeconomic status as a fundamental cause of reversed anemia risk among Yemeni youth
Source: PLoS One. 2026 Apr 30;21(4):e0348146. doi: 10.1371/journal.pone.0348146 (PMC13132244; doi:10.1371/journal.pone.0348146)
Supplement: S5 File — (DOCX) [file pone.0348146.s016.docx]

Supporting Information 5

COMPREHENSIVE DATA COLLECTION MANUAL

**The Healthy Smoker Paradox Study**

**1.0 STUDY OVERVIEW AND OBJECTIVES**

**1.1 Research Purpose**

This manual provides standardized procedures for data collection in the multi-center study "The Healthy Smoker Paradox: Socioeconomic Status as a Fundamental Cause of Reversed Anemia Risk among Yemeni Youth." The study aims to investigate the paradoxical relationship between smoking status and hematological parameters in the context of socioeconomic deprivation.

**1.2 Target Population**

- Yemeni university students aged 18-25 years

- Three participating universities in Southern Yemen

- Balanced representation across faculties (Medical Sciences, Engineering, Humanities)

**2.0 PARTICIPANT RECRUITMENT**

**2.1 Eligibility Criteria**

**Inclusion Criteria:**

- Currently enrolled undergraduate student

- Age 18-25 years

- Willing to provide blood sample

- Ability to provide informed consent

**Exclusion Criteria:**

- Pregnancy (self-reported)

- Known hematological disorders

- Blood transfusion in past 3 months

- Current anticoagulant therapy

- Inability to complete questionnaire

**2.2 Recruitment Procedures**

1. University Coordination: Obtain official permission from university administrations

2. Classroom Announcements: Brief presentations in selected classes

3. Poster Campaign: Study advertisements in common areas

4. Stratified Sampling: Ensure proportional representation across faculties

5. Screening: Verify eligibility before enrollment

**3.0 DATA COLLECTION PROCEDURES**

**3.1 Pre-Collection Preparation**

**Required Materials:**

- Numbered participant ID cards

- Structured questionnaires (English/Arabic)

- Informed consent forms

- Privacy screens for confidential responses

- Blood collection supplies (see Laboratory Manual)

**Staff Training:**

- 2-day training workshop for all data collectors

- Standardized explanation of study purpose

- Practice sessions for questionnaire administration

- Ethical considerations and confidentiality protocols

**3.2 Questionnaire Administration**

**Step-by-Step Protocol:**

**1. Welcome and Introduction (5 minutes)**

- Greet participant warmly

- Explain study purpose in standardized language

- Emphasize voluntary participation and confidentiality

**2. Informed Consent Process (10 minutes)**

- Review consent form section by section

- Allow time for questions

- Obtain signature and date

- Provide copy to participant

**3. Questionnaire Administration (25-30 minutes)**

- Read questions clearly and neutrally

- Allow participant to respond without interruption

- Maintain privacy during sensitive questions

- Clarify questions if needed, but avoid leading

**4. Anthropometric Measurements (5 minutes)**

- Height: Without shoes, heels together

- Weight: Light clothing, calibrated scale

- BMI: Calculate immediately (weight kg / height m²)

**3.3 Blood Collection Coordination**

**Scheduling**:

- Schedule blood collection within 48 hours of questionnaire

- Ensure overnight fasting (8-12 hours)

- Provide fasting instructions clearly

- Confirm appointment day before

**Logistics**:

- Coordinate with laboratory team

- Prepare participant identification

- Ensure comfortable waiting area

- Provide light snack after blood collection

**4.0 QUALITY ASSURANCE IN DATA COLLECTION**

**4.1 Data Quality Checks**

**Daily Procedures:**

- Verify completeness of all forms

- Check for logical inconsistencies

- Ensure proper participant identification

- Document any deviations from protocol

**Weekly Procedures:**

- Random audit of 10% completed questionnaires

- Cross-check data entry accuracy

- Review consent form compliance

- Address any staff training needs

**4.2 Problem Resolution**

**Common Issues and Solutions:**

- Participant discomfort: Pause interview, offer water, resume when ready

- Language barriers: Use bilingual staff or approved translators

- Sensitive questions: Emphasize confidentiality, allow skipping if uncomfortable

- Technical issues: Document and consult study coordinator

**5.0 ETHICAL CONSIDERATIONS**

**5.1 Confidentiality Protocols**

- Store completed forms in locked cabinets

- Use participant ID numbers instead of names

- Separate consent forms from questionnaire data

- Secure electronic data with password protection

- Destroy identifying information after study completion

**5.2 Participant Safety**

- Trained phlebotomists for blood collection

- Emergency protocol for vasovagal reactions

- Referral system for abnormal results

- Psychological support resources available

**5.3 Beneficence and Justice**

- Provide small compensation for time (equivalent to $5)

- Offer individual hematological results to participants

- Ensure equitable access across socioeconomic groups

- Share aggregate findings with participating universities

**6.0 DOCUMENTATION AND RECORD KEEPING**

**6.1 Required Documentation**

**Daily Log:**

- Number of participants enrolled

- Any protocol deviations

- Equipment malfunctions

- Participant concerns or complaints

**Study Master File:**

- Training certificates for all staff

- Ethical approval documents

- Protocol amendments

- Quality assurance reports

**6.2 Data Security**

**Physical Security:**

- Locked filing cabinets for paper records

- Restricted access to data storage areas

- Secure disposal of sensitive documents

**Digital Security:**

- Password-protected databases

- Regular backup procedures

- Encryption for data transfer

- Access logs for electronic files

**7.0 MONITORING AND SUPERVISION**

**7.1 Site Supervision**

**Weekly Monitoring:**

- Random observation of data collection

- Review of completed questionnaires

- Verification of consent procedures

- Equipment calibration checks

**Monthly Reporting:**

- Enrollment progress by stratum

- Protocol adherence metrics

- Participant satisfaction feedback

- Adverse event documentation

**7.2 Quality Indicators**

**Performance Metrics:**

- Questionnaire completion rate: Target >95%

- Blood sample success rate: Target >98%

- Participant satisfaction: Target >90%

- Protocol deviation rate: Target <5%

**8.0 PROTOCOL DEVIATIONS AND ADVERSE EVENTS**

**8.1 Reporting Procedures**

**Minor Deviations:**

- Document in daily log

- Report to site coordinator weekly

- Implement corrective actions

**Major Deviations/Adverse Events:**

- Immediate reporting to principal investigator

- Documentation on incident report form

- Review by ethics committee if required

- Participant follow-up as needed

**8.2 Corrective Actions**

**Training Gaps:**

- Additional training sessions

- Mentoring by experienced staff

- Performance monitoring

**Equipment Issues:**

- Immediate repair or replacement

- Validation of alternative methods

- Documentation of impact on data quality
