# Supplementary material for: The Healthy Smoker Paradox: Socioeconomic status as a fundamental cause of reversed anemia risk among Yemeni youth
Source: PLoS One. 2026 Apr 30;21(4):e0348146. doi: 10.1371/journal.pone.0348146 (PMC13132244; doi:10.1371/journal.pone.0348146)
Supplement: S6 File — (DOCX) [file pone.0348146.s017.docx]

**Supporting information 6**

**COMPREHENSIVE INSTRUMENT CALIBRATION PROTOCOLS**

**1.0 HEMATOLOGY ANALYZER CALIBRATION (Mindray BC-3000 Plus)**

**1.1 Daily Calibration Procedures**

**Pre-Calibration Checks:**

- Verify analyzer is in ready state

- Check reagent levels and expiration dates

- Ensure waste container has adequate capacity

- Confirm proper system temperature (18-25°C)

**Calibration Steps:**

**1. System Prime:**

- Run system prime procedure

- Check for air bubbles in fluidic system

- Verify waste flow

**2. Background Check:**

- Perform background count using diluent

- Accept if WBC < 0.2, RBC < 0.02, HGB < 0.1, PLT < 10

- Document results in calibration log

**3. Calibrator Analysis:**

- Use manufacturer-provided calibrators (3 levels)

- Run each level in duplicate

- Accept if within manufacturer's specified ranges

**1.2 Monthly Performance Verification**

**Precision Testing:**

- Run 20 consecutive measurements of normal control

- Calculate mean, standard deviation, and CV%

- Accept if CV% meets manufacturer specifications:

- WBC: CV ≤ 3.0%

- RBC: CV ≤ 2.0%

- HGB: CV ≤ 2.0%

- PLT: CV ≤ 5.0%

**Linearity Verification:**

- Prepare serial dilutions of high-value sample

- Analyze across measuring range

- Verify linear regression R² > 0.975

- Document deviation from linearity

**1.3 Quarterly Maintenance**

**Complete System Maintenance:**

- Replace worn components per schedule

- Clean optical systems and detectors

- Update software if available

- Performance verification after maintenance

**2.0 COAGULATION ANALYZER CALIBRATION (BA-88A Semi-automated)**

**2.1 Daily Calibration Procedures**

**Temperature Verification:**

- Confirm heating block temperature: 37.0°C ± 0.5°C

- Check water bath temperature if used

- Document temperature readings

**Photometric System Check:**

- Verify detector baseline with blank

- Check light source intensity

- Confirm proper wavelength calibration

**2.2 Reagent Calibration**

**PT Calibration:**

- Use manufacturer's calibration plasma

- Establish mean normal value for each reagent lot

- Verify with secondary reference material

- Document ISI (International Sensitivity Index)

**APTT Calibration:**

- Establish reference range with local population

- Verify with commercial control materials

- Document mean normal value

**2.3 Monthly Quality Assessment**

**Precision Study:**

- Run normal and abnormal controls in duplicate daily for 10 days

- Calculate within-run and between-run precision

- Accept if CV% ≤ 5.0% for both PT and APTT

**Accuracy Verification:**

- Participate in external quality assurance program

- Verify results against assigned values

- Investigate and correct any biases

**3.0 ANTHROPOMETRIC EQUIPMENT CALIBRATION**

**3.1 Digital Scale Calibration**

**Daily Verification:**

- Check with standard weights (1kg, 5kg, 10kg)

- Verify accuracy within ± 0.1 kg

- Document any adjustments

**Monthly Calibration:**

- Full calibration with certified weights

- Check across measurement range

- Service if outside specifications

**3.2 Stadiometer Calibration**

**Weekly Verification:**

- Check with fixed measurement standard

- Verify accuracy within ± 0.5 cm

- Ensure proper vertical alignment

**Quarterly Maintenance:**

- Check for wear on moving parts

- Verify stability of base

- Clean measurement surface

**4.0 DOCUMENTATION AND RECORDS**

**4.1 Calibration Logs**

**Required Entries:**

- Date and time of calibration

- Equipment identification number

- Calibrator lot numbers and expiration

- Pre- and post-calibration values

- Technician signature

- Any corrective actions taken

**4.2 Maintenance Records**

**Scheduled Maintenance:**

- Preventive maintenance schedule

- Parts replacement records

- Service engineer reports

- Performance verification after service

**4.3 Quality Control Charts**

**Levey-Jennings Charts:**

- Daily control values for all parameters

- Calculation of means and standard deviations

- Visual inspection for trends or shifts

- Documentation of any rule violations

**5.0 CORRECTIVE ACTIONS**

**5.1 Out-of-Range Results**

**Immediate Actions:**

- Repeat calibration with fresh reagents

- Check for technical errors

- Verify proper sample handling

- Consult troubleshooting guide

**Escalation Procedures:**

- Notify laboratory supervisor

- Document incident in quality log

- Implement temporary alternative methods if needed

- Arrange for service if problem persists

5.2 Preventive Measures

**Regular Monitoring:**

- Review calibration trends monthly

- Compare performance across instruments

- Update procedures based on performance data

- Staff training on recognition of instrument problems

---

**CALIBRATION PROTOCOL VERSION: 2.0**
