# Supplementary material for: The Healthy Smoker Paradox: Socioeconomic status as a fundamental cause of reversed anemia risk among Yemeni youth
Source: PLoS One. 2026 Apr 30;21(4):e0348146. doi: 10.1371/journal.pone.0348146 (PMC13132244; doi:10.1371/journal.pone.0348146)
Supplement: S7 File — (DOCX) [file pone.0348146.s018.docx]

**Supporting Information 7**

**DETAILED STATISTICAL ANALYSIS PLAN**

**The Healthy Smoker Paradox Study**

**1.0 STUDY HYPOTHESES**

**1.1 Primary Hypotheses**

1. Non-smokers will have significantly higher odds of abnormal hemoglobin levels compared to current smokers, after adjusting for age, gender, and BMI.

2. Non-smokers will have significantly higher odds of abnormal MCHC compared to current smokers, after adjusting for age, gender, and BMI.

**1.2 Secondary Hypotheses**

1. The association between smoking status and hematological abnormalities will be mediated by nutritional status.

2. The paradoxical association will be stronger among participants from lower socioeconomic strata.

3. The association will be modified by gender, with stronger effects in female participants.

**2.0 SAMPLE SIZE JUSTIFICATION**

**2.1 Power Calculation**

Parameters:

- Alpha (α) = 0.05 (two-tailed)

- Power (1-β) = 0.80

- Effect size (odds ratio) = 2.5 (based on pilot data)

- Smoking prevalence = 24% (based on university statistics)

- Anemia prevalence in non-smokers = 34% (based on pilot data)

Calculation:

- Required sample size: 552 participants

- Target enrollment: 600 participants (accounting for 8% attrition)

- Allocation ratio: 1:3 (smokers:non-smokers)

**2.2 Subgroup Power**

Stratified Analyses:

- Gender stratification: 350 males, 250 females (adequate power)

- SES tertiles: 200 per tertile (adequate power)

- University sites: 200 per site (adequate power)

**3.0 PRIMARY ANALYSIS PLAN**

**3.1 Analysis Population**

Intent-to-Treat Principle:

- All enrolled participants with complete baseline data

- No imputation for primary exposure (smoking status)

- Complete case analysis for primary outcomes

Exclusion Criteria:

- Missing primary exposure data

- Missing primary outcome data

- Protocol violations affecting exposure assessment

**3.2 Primary Statistical Models**

Model 1: Unadjusted Association

- Logistic regression: Outcome ~ Smoking status

Model 2: Minimally Adjusted

- Logistic regression: Outcome ~ Smoking status + Age + Gender

Model 3: Fully Adjusted

- Logistic regression: Outcome ~ Smoking status + Age + Gender + BMI + University

**3.3 Model Assumptions Checking**

Logistic Regression Assumptions:

- Linearity of continuous covariates with log odds

- Absence of multicollinearity (VIF < 5)

- Adequate cell sizes for categorical variables

- Independence of observations

**4.0 SECONDARY ANALYSIS PLAN**

**4.1 Mediation Analysis**

Framework: Causal mediation analysis using counterfactual approach

Software: R mediation package with bootstrapping

Models:

- Mediator model: Nutrition ~ Smoking + Covariates

- Outcome model: Hematology ~ Smoking + Nutrition + Covariates

Bootstrap samples: 5000

Confidence intervals: Bias-corrected 95% CI

**4.2 Effect Modification Analysis**

Interaction Terms:

- Smoking × Socioeconomic status

- Smoking × Gender

- Smoking × University site

Assessment: Likelihood ratio tests for interaction terms

Stratified analysis: If significant interaction (p < 0.10)

**4.3 Sensitivity Analyses**

Multiple Imputation:

- Method: Predictive mean matching

- Number of imputations: 5

- Variables imputed: BMI, SES index, dietary diversity

Propensity Score Methods:

- Matching: 1:1 nearest neighbor

- Weighting: Inverse probability of treatment weights

- Covariates: Age, gender, BMI, SES, university

E-value Analysis:

- Quantify unmeasured confounding strength

- Calculate for point estimates and confidence limits

**5.0 EXPLORATORY ANALYSES**

**5.1 Dose-Response Relationships**

Smoking Intensity:

- Categorical analysis: 1-5, 6-10, 11-20, >20 cigarettes/day

- Continuous analysis: Per cigarette increase

- Test for linear trend

**5.2 Additional Hematological Parameters**

Secondary Outcomes:

- Platelet count abnormalities

- Coagulation parameter abnormalities

- Red blood cell indices

- White blood cell count

**5.3 Behavioral Covariates**

Additional Adjustments:

- Khat chewing frequency

- Sleep duration patterns

- Physical activity levels

- Dietary diversity scores

**6.0 MULTIPLE TESTING CONSIDERATIONS**

**6.1 Primary Outcomes**

No adjustment: Two primary hypotheses specified a priori

Interpretation: Family-wise error rate controlled for primary aims

**6.2 Secondary Outcomes**

False Discovery Rate: Benjamini-Hochberg procedure

Threshold: FDR < 0.05 for secondary hypothesis testing

**6.3 Exploratory Analyses**

No adjustment: Clearly labeled as exploratory

Interpretation: Hypothesis-generating only

**7.0 MISSING DATA HANDLING**

**7.1 Missing Data Patterns**

Assessment: Little's MCAR test

Documentation: Pattern and mechanism of missingness

**7.2 Handling Strategies**

Primary analysis: Complete case

Sensitivity analysis: Multiple imputation

Exclusion: If >10% data missing for critical variables

**Enhanced Missing Data Protocol:**

Missing data patterns were assessed using Little's MCAR test (χ² = 15.23, p = 0.234), supporting the missing completely at random assumption. Primary analyses used complete cases given the low missingness (<5% for all primary variables). Sensitivity analyses with multiple imputation (m=5 datasets) using predictive mean matching produced nearly identical results, confirming robustness to missing data assumptions.

For the primary exposure (smoking status) and outcomes (hematological parameters), complete case analysis was employed given the critical nature of these variables. For covariates with missing data (SES index 4%, dietary diversity 3%), multiple imputation was used in sensitivity analyses with minimal impact on effect estimates.

**8.0 SOFTWARE AND IMPLEMENTATION**

**8.1 Statistical Software**

Primary: R version 4.3.1

Secondary: IBM SPSS Statistics 28

Specialized packages:

- mediation: Causal mediation analysis

- MatchIt: Propensity score matching

- EValue: Sensitivity analysis

- mice: Multiple imputation

**8.2 Code Management**

Version control: Git repository

Documentation: R Markdown for reproducible reports

Validation: Independent code review by second statistician

**9.0 REPORTING STANDARDS**

**9.1 Adherence to Guidelines**

STROBE: Strengthening the Reporting of Observational Studies

TRIPOD: Transparent Reporting of multivariable prediction model

**9.2 Results Presentation**

Primary results: Adjusted odds ratios with 95% confidence intervals

Mediation results: Direct, indirect, and total effects

Sensitivity results: Comparison across methods

**10.0 ANALYSIS TIMELINE**

**10.1 Phased Approach**

Phase 1: Descriptive statistics and data quality checks

Phase 2: Primary hypothesis testing

Phase 3: Secondary and sensitivity analyses

Phase 4: Exploratory and subgroup analyses

**10.2 Deliverables**

Statistical report: Comprehensive analysis document

Publication tables: Formatted results tables

Supporting Information s: Extended analyses and code

---

SAP VERSION: 2.0
