# Supplementary material for: The Healthy Smoker Paradox: Socioeconomic status as a fundamental cause of reversed anemia risk among Yemeni youth
Source: PLoS One. 2026 Apr 30;21(4):e0348146. doi: 10.1371/journal.pone.0348146 (PMC13132244; doi:10.1371/journal.pone.0348146)
Supplement: S8 File — (DOCX) [file pone.0348146.s019.docx]

**File S8: DATA CLEANING PROTOCOL
The Healthy Smoker Paradox Study
Version: 2.0
Date: March 2026**

# 1.0 INITIAL DATA IMPORT AND INSPECTION

# Read raw data

df_raw <- read.csv("File_S1_Raw_Data_Original.csv", stringsAsFactors = FALSE)

# Display basic information

cat("=== DATA CLEANING PROTOCOL ===\n")

cat("Date:", Sys.time(), "\n")

cat("Initial dimensions:", dim(df_raw), "\n")

cat("Variable names:", names(df_raw), "\n")

# Check for duplicate IDs

duplicate_ids <- df_raw$id[duplicated(df_raw$id)]

if (length(duplicate_ids) > 0) {

cat("WARNING: Duplicate IDs found:", length(duplicate_ids), "\n")

} else {

cat("No duplicate IDs found.\n")

}

# 2.0 REMOVE IDENTIFIERS (ANONYMIZATION)

# Remove the ID column as per journal requirements

df_clean <- df_raw[, !names(df_raw) %in% c("id", "name", "initials", "phone", "email")]

cat("\nRemoved identifier columns. New dimensions:", dim(df_clean), "\n")

# 3.0 HANDLE MISSING DATA

# Identify missing values

missing_summary <- data.frame(

Variable = names(df_clean),

Missing_n = sapply(df_clean, function(x) sum(is.na(x))),

Missing_pct = sapply(df_clean, function(x) mean(is.na(x)) * 100)

)

print(missing_summary)

# List variables with missing data

variables_with_missing <- missing_summary$Variable[missing_summary$Missing_n > 0]

cat("\nVariables with missing data:",

paste(variables_with_missing, collapse = ", "), "\n")

# Perform Little's MCAR test (if mice package is available)

if (require(mice, quietly = TRUE)) {

if (length(variables_with_missing) > 1) {

mcar_test <- LittleMCAR(df_clean[, variables_with_missing])

print(mcar_test)

}

}

# 4.0 DATA VALIDATION AND RANGE CHECKS

# Age range check

age_valid <- df_clean$age >= 18 & df_clean$age <= 25

if (all(age_valid, na.rm = TRUE)) {

cat("\nAge range: All values within 18-25 years.\n")

} else {

cat("\nWARNING: Invalid ages found:",

sum(!age_valid, na.rm = TRUE), "records\n")

df_clean$age[!age_valid] <- NA

}

# BMI range check

bmi_valid <- df_clean$bmi >= 12 & df_clean$bmi <= 45

if (!all(bmi_valid, na.rm = TRUE)) {

cat("WARNING: Invalid BMI values found:",

sum(!bmi_valid, na.rm = TRUE), "records\n")

}

# Hemoglobin range check

hb_valid <- df_clean$hb >= 6 & df_clean$hb <= 20

if (!all(hb_valid, na.rm = TRUE)) {

cat("WARNING: Invalid hemoglobin values found:",

sum(!hb_valid, na.rm = TRUE), "records\n")

}

# 5.0 RECODE CATEGORICAL VARIABLES

# Smoking status recoding

df_clean$smoking_status <- factor(df_clean$smoking,

levels = c("Never", "Former", "Current"),

labels = c("Never-smoker", "Former smoker", "Current smoker"))

# Gender recoding

df_clean$gender <- factor(df_clean$gender,

levels = c("Male", "Female"))

# Khat chewing recoding

df_clean$khat <- factor(df_clean$khat,

levels = c("Never", "Occasionally", "Weekly", "Daily"))

# Sleep duration recoding

df_clean$sleep_cat <- factor(df_clean$sleep_cat,

levels = c("<3h", "4-7h", "8-11h"))

# 6.0 CREATE DERIVED VARIABLES

# Create binary smoking indicator (Current smoker vs Never smoker)

df_clean$is_smoker <- ifelse(df_clean$smoking_status == "Current smoker", 1, 0)

df_clean$never_smoker <- ifelse(df_clean$smoking_status == "Never-smoker", 1, 0)

# Create binary anemia variable

df_clean$anemia <- df_clean$hb_abn

# Create binary MCHC abnormal variable

df_clean$mchc_abnormal <- df_clean$mchc_abn

# Create binary PT abnormal variable

df_clean$pt_abnormal <- df_clean$pt_abn

# Create binary APTT abnormal variable

df_clean$ptt_abnormal <- df_clean$ptt_abn

# Create binary platelet abnormal variable

df_clean$plt_abnormal <- df_clean$plt_abn

# 7.0 HANDLE OUTLIERS

# Function to detect outliers using IQR method

detect_outliers <- function(x) {

q1 <- quantile(x, 0.25, na.rm = TRUE)

q3 <- quantile(x, 0.75, na.rm = TRUE)

iqr <- q3 - q1

lower_bound <- q1 - 1.5 * iqr

upper_bound <- q3 + 1.5 * iqr

return(x < lower_bound | x > upper_bound)

}

# Identify outliers in continuous variables

continuous_vars <- c("age", "bmi", "hb", "mchc", "mcv", "plt", "wbc", "pt", "ptt")

outlier_flags <- data.frame(sapply(df_clean[, continuous_vars], detect_outliers))

# Count outliers per variable

cat("\nOutlier counts:\n")

colSums(outlier_flags, na.rm = TRUE)

# 8.0 HANDLE INCONSISTENCIES

# Check for logical inconsistencies

# e.g., Former smokers with current smoking status

inconsistent_former <- df_clean$smoking == "Former" & df_clean$cigarettes_per_day > 0

if (any(inconsistent_former, na.rm = TRUE)) {

cat("\nWARNING: Inconsistent former smoker records:",

sum(inconsistent_former, na.rm = TRUE), "\n")

}

# Check for missing hemoglobin with abnormal flag

hb_missing_flag <- is.na(df_clean$hb) & df_clean$hb_abn == 1

if (any(hb_missing_flag, na.rm = TRUE)) {

cat("WARNING: Abnormal flag with missing hemoglobin value:",

sum(hb_missing_flag, na.rm = TRUE), "\n")

}

# 9.0 STANDARDIZE VARIABLES

# Standardize continuous variables for analysis

df_clean$age_std <- scale(df_clean$age)

df_clean$bmi_std <- scale(df_clean$bmi)

df_clean$hb_std <- scale(df_clean$hb)

# 10.0 EXPORT CLEANED DATASET

# Remove columns with only missing data

df_clean <- df_clean[, colSums(is.na(df_clean)) < nrow(df_clean)]

# Final dataset dimensions

cat("\n=== FINAL DATASET ===\n")

cat("Dimensions:", dim(df_clean), "\n")

cat("Variables:", paste(names(df_clean), collapse = ", "), "\n")

# Export to CSV

write.csv(df_clean, "File_S1_Raw_Data_Anonymized.csv", row.names = FALSE)

# 11.0 DATA QUALITY REPORT

# Generate data quality report

quality_report <- list(

Date = Sys.time(),

Original_N = nrow(df_raw),

Final_N = nrow(df_clean),

Variables_Removed = setdiff(names(df_raw), names(df_clean)),

Missing_Data_Summary = missing_summary,

Outlier_Summary = colSums(outlier_flags, na.rm = TRUE),

Validation_Status = "PASSED"

)

# Save quality report

saveRDS(quality_report, "Data_Quality_Report.rds")

# Print summary

cat("\n=== DATA QUALITY REPORT ===\n")

cat("Original N:", quality_report$Original_N, "\n")

cat("Final N:", quality_report$Final_N, "\n")

cat("Variables removed:", paste(quality_report$Variables_Removed, collapse = ", "), "\n")

# 12.0 CLEANING LOG

cleaning_log <- data.frame(

Step = c("Import", "Remove IDs", "Missing handling", "Range checks",

"Recoding", "Derived variables", "Outlier detection", "Export"),

Timestamp = rep(Sys.time(), 8),

Records_Affected = c(nrow(df_raw), nrow(df_clean),

sum(missing_summary$Missing_n),

sum(!age_valid | !bmi_valid, na.rm = TRUE),

nrow(df_clean), nrow(df_clean),

sum(colSums(outlier_flags, na.rm = TRUE)),

nrow(df_clean)),

Status = c("OK", "OK", "OK", "OK", "OK", "OK", "OK", "OK")

)

print(cleaning_log)

write.csv(cleaning_log, "Cleaning_Log.csv", row.names = FALSE)

# END OF DATA CLEANING PROTOCOL

cat("\n=== CLEANING COMPLETE ===\n")

cat("Cleaned data saved to: File_S1_Raw_Data_Anonymized.csv\n")

cat("Quality report saved to: Data_Quality_Report.rds\n")

cat("Cleaning log saved to: Cleaning_Log.csv\n")
