# Supplementary material for: The Healthy Smoker Paradox: Socioeconomic status as a fundamental cause of reversed anemia risk among Yemeni youth
Source: PLoS One. 2026 Apr 30;21(4):e0348146. doi: 10.1371/journal.pone.0348146 (PMC13132244; doi:10.1371/journal.pone.0348146)
Supplement: Attachment — Submitted filename: Response to Editor.pdf [file pone.0348146.s021.pdf]

Response to Editor:

Dear Dr. Florie Lacorte Canales,

Thank you for your editorial feedback on our manuscript PONE-D-25-63271, "The Healthy Smoker Paradox: Socioeconomic Status as a Fundamental Cause of Reversed Anemia Risk among Yemeni Youth." We appreciate the opportunity to revise our submission in accordance with PLOS ONE's submission guidelines.

Point 1: Please report in the Methods section the day, month, and year of the start and end of the recruitment period for this study in DD/MM/YYYY format.

Response: We have updated the Methods section under "Study Design and Participants" to explicitly state the precise recruitment period in the requested format. The revised text now reads:

"A multi-center, cross-sectional study was conducted between 15/01/2025 and 2025/05/30 at three major universities in Southern Yemen (University of Science and Technology - Aden, University of Lahej, and Aden Gulf International University - Al-Dhale)."

This revision ensures full transparency regarding the study timeline and complies with the journal's submission requirements.

Thank you for your guidance. We have uploaded the revised manuscript file and approved the rebuilt PDF accordingly. We look forward to the next stage of the editorial process.

Sincerely,

Dr. Radfan Saleh Abdullah

On behalf of all co-authors
