# Supplementary material for: The Healthy Smoker Paradox: Socioeconomic status as a fundamental cause of reversed anemia risk among Yemeni youth
Source: PLoS One. 2026 Apr 30;21(4):e0348146. doi: 10.1371/journal.pone.0348146 (PMC13132244; doi:10.1371/journal.pone.0348146)
Supplement: Attachment — Submitted filename: point to point response.pdf [file pone.0348146.s022.pdf]

Response To Editor and Reviewers Comments

Manuscript ID: PONE-D-25-63271

Title: The Healthy Smoker Paradox: Socioeconomic Status as a Fundamental Cause of Reversed Anemia Risk among Yemeni Youth

Journal: PLOS ONE

Date: March 26, 2026

Dear Dr. Al-Nimer and Esteemed Reviewers,

We would like to express our sincere gratitude for the opportunity to revise our manuscript entitled "The Healthy Smoker Paradox: Socioeconomic Status as a Fundamental Cause of Reversed Anemia Risk among Yemeni Youth." We are deeply appreciative of the thoughtful and constructive feedback provided by the Academic Editor and the three reviewers. Their insightful comments have been instrumental in strengthening our work, and we believe the revised manuscript is substantially improved as a result of their careful review.

In the following sections, we provide a detailed, point-by-point response to all comments raised. We have addressed each concern with appropriate revisions to the manuscript, and all changes have been highlighted using the track changes feature. We have also taken this opportunity to enhance the technical quality of our figures by converting them from PNG to TIFF format, ensuring compliance with PLOS ONE's publication requirements. We trust that our revisions adequately address all concerns and that the manuscript now meets the high standards of PLOS ONE.

Response to the Academic Editor

We thank the Academic Editor, Dr. Marwan Salih Al-Nimer, for overseeing the review process and for the invitation to submit a revised manuscript. We have carefully addressed all journal requirements as outlined.

Journal Requirement 1: PLOS ONE Style Requirements

We have thoroughly reviewed the PLOS ONE style templates and have reformatted our manuscript accordingly. All sections now adhere to the journal's formatting guidelines, including proper heading hierarchy, line spacing, and reference formatting. File naming conventions have also been standardized to meet journal specifications.

Journal Requirement 2: Data Availability Statement

We have updated the Data Availability Statement in the submission form. The complete statement now reads: "Due to the sensitive nature of the data collected from human participants and the potential for identification given the small population size, the raw data cannot be made publicly available to protect participant privacy. An anonymized dataset with all direct identifiers removed is available from

the corresponding author (Radfan Saleh Abdullah, r.saleh@ust.edu) upon reasonable request and following approval from the institutional ethics committee. All other relevant data are within the manuscript and its Supporting Information files."

Journal Requirement 3: Ethics Statement Location

We have verified that the ethics statement appears exclusively in the Methods section of the manuscript. Any duplicate statements elsewhere have been removed. The ethics statement now reads: "The study protocol received full approval from the Research Ethics Committee of the Faculty of Medicine and Health Sciences, University of Science and Technology (Approval No. MEC/AD087), as well as from the ethics committees of all participating institutions. The study was conducted in accordance with the ethical principles of the Declaration of Helsinki. Prior to participation, all subjects received comprehensive information about the study objectives and procedures. Written informed consent was obtained from each participant. Confidentiality and anonymity were strictly maintained throughout all stages of the research, including data collection, analysis, and publication."

Journal Requirement 4: Corrupted Supporting Information Files

We acknowledge that the following files were previously unreadable: File S3 COMPREHENSIVE R ANALYSIS SCRIPT.R, File S8.R, and File_S3_Analysis scripts.R. We have completely rewritten these files with comprehensive documentation and proper formatting. The revised files have been tested and verified to function correctly. They have been renamed for clarity and are now uploaded as:

· File_S3_R_Analysis_Script.R (formerly File S3 COMPREHENSIVE R ANALYSIS SCRIPT.R)

· File_S8_Data_Cleaning_Protocol.R (formerly File S8.R)

· File_S3A_Supplementary_Analysis_Scripts.R (formerly File_S3_Analysis scripts.R)

Journal Requirement 5: Anonymization of Participant Data

We thank the editor for highlighting this important ethical consideration. We have created a fully anonymized version of the dataset with the following modifications: (1) the participant ID column has been removed entirely; (2) no names, initials, or contact information were ever present in the dataset; and (3) the dataset now contains only non-identifying demographic, behavioral, and laboratory variables. The anonymized dataset has been re-uploaded as File_S1_Raw_Data_Anonymized.csv. In accordance with participant consent and local regulations, this dataset is shared under restricted access and is available upon reasonable request from the corresponding author.

Journal Requirement 6: Review of Suggested Citations

We have carefully reviewed all references suggested by the reviewers. All cited references are relevant to our study and have been retained. Duplicate references 19 and 20 have been corrected as noted in our response to Reviewer #3.

Additional Editor Comments: Major Revision

We have undertaken a major revision of the manuscript, addressing all points raised by the reviewers. The key areas of revision include: tempering causal language throughout the manuscript; clarifying the classification of smoking groups and excluding former smokers from the primary analysis; revising the sample size calculation; expanding the limitations section to address unmeasured confounders; adding a discussion of coagulation parameter findings; correcting typographical errors; revising Table 1, Table 2, and Table 3 to reduce redundancy and improve completeness; and ensuring all figures meet PLOS ONE technical requirements. We have also converted all figures from PNG to TIFF format to ensure compliance with the journal's specifications for high-resolution images.

Response to Reviewer #1: Dr. Aisha Al Khinji

We are grateful to Reviewer #1 for recognizing the novelty and theoretical grounding of our work. The constructive criticisms have helped us refine our interpretations and strengthen the manuscript's scientific rigor.

Comment 1: Cross-Sectional Design and Causal Interpretation

The reviewer noted that our cross-sectional design limits causal inference and that our interpretation overstates causal conclusions. We agree with this important point. Throughout the revised manuscript, we have systematically replaced causal language with language that appropriately reflects the associative nature of our findings. Specifically, we have changed "document" to "suggest," "serves as" to "may serve as," "demonstrates" to "is consistent with," and "provides compelling evidence" to "provides evidence consistent with the hypothesis." These changes have been implemented in the Abstract, Introduction, Results, Discussion, and Conclusion sections. We have also added explicit acknowledgment of the cross-sectional limitation in the Strengths and Limitations section, noting that definitive causal conclusions about temporal sequence cannot be drawn from our data.

Comment 2: Residual Confounding

The reviewer raised concerns about residual confounding, particularly from nutritional, infectious, and socioeconomic factors. We have substantially expanded the Limitations section to address these concerns explicitly. We now acknowledge the absence of direct micronutrient biomarkers (ferritin, vitamin B12, folate), the lack of measurement of parasitic infections (schistosomiasis, malaria), the absence of systemic inflammation markers, and the limitations of our nutritional assessment. Importantly, we have retained and highlighted our E-value sensitivity analysis, which demonstrates that an unmeasured confounder would need to be associated with both smoking status and anemia by risk ratios of 4.32-fold each to fully explain the observed association—a strength that exceeds most known anemia risk factors in this population context.

Comment 3: Smoking as a Proxy for SES

The reviewer noted that our assumption that smoking serves as a proxy for SES is plausible but not fully demonstrated. We agree and have revised our language throughout to reflect that this is an interpretation consistent with our findings, not a definitive demonstration. We have added cautionary language in the Discussion and Conclusion sections, emphasizing that our interpretation is supported by the mediation analysis, interaction effects, and E-value sensitivity analyses, but remains inferential given the cross-sectional design.

Comment 4: Generalizability to University Populations

The reviewer correctly noted that generalizability is limited to university populations. We have added explicit acknowledgment of this limitation in the Strengths and Limitations section, stating that the findings are specific to university students and may not generalize to the broader Yemeni youth population. We have also added a justification in the Introduction explaining why university students were selected as a stable, accessible population for initial hypothesis testing before generalization to the broader population.

Response to Reviewer #2: Shadrack Barffour Awuah

We are deeply grateful to Reviewer #2 for the detailed and constructive feedback. The methodological points raised have significantly improved the rigor of our manuscript.

Comment 1: Overstated Language

The reviewer recommended replacing overstated words such as "powerful," "compelling," "striking," and "dramatically" with more neutral terminology. We have conducted a comprehensive review of the manuscript and replaced these terms throughout. Specific changes include: "powerful and statistically striking paradox" now reads "unexpected and statistically robust association"; "compelling demonstration" now reads "findings consistent with"; "dramatically higher odds" now reads "substantially higher odds"; and "stark differences" now reads "notable differences." These changes have been implemented across all sections of the manuscript.

Comment 2: Reframing the Hypothesis

The reviewer correctly noted that our hypothesis was written as a conclusion rather than a testable statement. We have revised the hypothesis in the Introduction to reflect appropriate caution. The original text stating "smoking serves as a proxy for higher SES" has been changed to "smoking may function as a proxy for relative socioeconomic advantage in this context." We have similarly revised the accompanying hypothesis about mediation and effect modification to include appropriate conditional language.

Comment 3: Justification for University Student Selection

The reviewer requested a short justification for why university students were selected and why they remain relevant in a conflict setting. We have added the following text to the Introduction: "University students were selected as they represent a stable, accessible population that allows for standardized data collection across multiple sites, providing a controlled setting to test our hypothesis before generalization to the broader, more heterogeneous youth population. Despite being enrolled in higher education, this population remains highly vulnerable to the country's economic and nutritional crises, as university students often face food insecurity and financial constraints similar to non-student youth."

Comment 4: Clarification of Smoking Classification

This was a critical methodological issue. The reviewer noted an inconsistency between our Methods statement (comparing current smokers vs. never-smokers) and our Results statement (including former smokers in the non-smoker group). We have corrected this issue comprehensively. First, in the Methods section, we now explicitly state that former smokers were excluded from the primary analysis due to their biological and behavioral differences from never-smokers. Second, in the Results section, we now report that after excluding former smokers (n=48, 8.0%), the analytic cohort comprised 144 current smokers (24.0%) and 408 never-smokers 68.0%)). Third, we have conducted a sensitivity analysis including former smokers in the non-smoker group to assess the robustness of our findings; the results remained consistent, though with slightly attenuated effect estimates. Fourth, we have created a separate dataset for former smokers and uploaded it as File_S1_Former_Smokers_Only.csv for transparency.

Comment 5: Sample Size Calculation

The reviewer noted that our original sample size calculation using $f^2 = 0.15$ was too generic and mismatched our analysis. We have revised the sample size calculation using the recommended parameters. The revised calculation now reads: "Based on pilot data from the same population, we assumed an anemia prevalence of approximately 34% among non-smokers and a smoking prevalence of 24%. To detect a minimum clinically relevant odds ratio of 2.5 for the association between smoking status and anemia with 80% power and a two-sided alpha of 0.05, the required sample size was calculated as 552 participants. Accounting for an anticipated 8% attrition or incomplete data, we targeted enrollment of 600 participants."

Comment 6: Table 3 Completeness

The reviewer noted that Table 3 appeared incomplete, showing only a sleep variable and seeming to select only significant results. We have completely revised Table 3 to present the full logistic regression models for both primary outcomes (abnormal hemoglobin and abnormal MCHC). The table now includes all covariates (age, gender, BMI, and university site) with their respective adjusted odds ratios, confidence

intervals, and p-values. The full model results are now transparently presented.

Comment 7: Definitive Language in Conclusion

The reviewer recommended avoiding definitive language such as "demonstrates," "confirms," or "proves" in the Conclusion. We have thoroughly revised the Conclusion section to use appropriate cautionary language. The original statement "This study provides compelling evidence" now reads "This study provides evidence consistent with the hypothesis." Similarly, "Our findings demonstrate" now reads "These findings suggest." All causal claims have been replaced with associative language.

Comment 8: Policy Recommendation Language

The reviewer advised using language that suggests the study informs priorities rather than dictates policies. We have revised all policy recommendations to reflect this. We now use phrases such as "may inform," "may help guide," "suggests the need for," and "support consideration of" throughout the policy implications section.

Response to Reviewer #3

We thank Reviewer #3 for recognizing the overall quality of our work and for providing specific, actionable suggestions that have helped us strengthen the manuscript.

Comment 1: Overstated Interpretation

The reviewer noted that our interpretation of findings was too strong for a cross-sectional study. We have addressed this concern comprehensively by replacing causal language with associative language throughout the manuscript. This includes changes in the Abstract, Introduction, Results, Discussion, and Conclusion sections. We have also added explicit statements acknowledging the cross-sectional limitation and emphasizing that our findings demonstrate association, not causation.

Comment 2: Unmeasured Factors Affecting Hemoglobin

The reviewer identified several important factors not fully measured in our study, including parasitic infections, menstrual blood loss, inflammation, and micronutrient deficiencies. We have expanded the Limitations section to explicitly acknowledge these factors. The revised text now reads: "Several important factors that could influence hemoglobin levels were not fully measured, including parasitic infections (such as schistosomiasis and malaria), menstrual blood loss patterns, systemic inflammation markers, and comprehensive micronutrient biomarkers (e.g., ferritin, vitamin B12, folate). These factors could potentially confound or mediate the observed associations and should be addressed in future studies."

Comment 3: Generalizability Limitations

The reviewer correctly noted that our study population consists only of university students, which may limit generalizability. We have added explicit acknowledgment

of this limitation, stating: "Second, the study population consists exclusively of university students, which may introduce selection bias and limits generalizability to the broader Yemeni youth population or other humanitarian settings. University students represent a distinct subgroup with potentially different socioeconomic characteristics and health behaviors compared to non-students of similar age."

Comment 4: Self-Reported Smoking Status

The reviewer noted that smoking status was primarily self-reported with limited biochemical validation, which may result in misclassification bias. We have added acknowledgment of this limitation, stating: "Fifth, smoking status was primarily self-reported, with biochemical validation (cotinine testing) implemented in only 10% of participants due to resource constraints. This may result in misclassification bias, particularly in subgroups where underreporting of smoking could occur due to social desirability. However, random sampling for validation and consistency across smoking intensity categories mitigate concerns about substantial exposure misclassification."

Comment 5: Mediation Analysis Interpretation

The reviewer advised that the mediation analysis should be interpreted carefully and presented as exploratory given the cross-sectional design. We have revised the presentation of mediation results throughout the manuscript to reflect their exploratory nature. In the Abstract, we changed "revealed that 38%" to "suggested that 38%." In the Results section, we added cautionary language. In the Discussion, we added: "Therefore, these mediation results should be considered exploratory and hypothesis-generating rather than definitive causal evidence."

Comment 6: Clarification of "Healthy Smoker Paradox" Term

The reviewer noted that the term "healthy smoker paradox" may be misunderstood and suggested clarifying that the findings do not indicate any protective effect of smoking. We have added a clarifying statement in the Introduction and Discussion: "It should be emphasized that the term 'healthy smoker paradox' is used here to describe the unexpected direction of association and does not imply any protective biological effect of tobacco smoking."

Comment 7: Data Availability Statement

We have clarified the Data Availability Statement to meet journal requirements, as detailed in our response to the Academic Editor.

Comment 8: Typographical and Formatting Errors

We have carefully proofread the entire manuscript and corrected all typographical errors. Specific corrections include: "pattern.The" to "pattern. The"; "institutions.Written" to "institutions. Written"; and "80%power" to "80% power."

Comment 9: Duplicate References

We have corrected the duplicate references. Reference 20 has been removed, and all subsequent references have been renumbered accordingly. The reference list now contains no duplicates.

Comment 10: Redundancy Between Table 1 and Table 2

We thank the reviewer for this observation. We have revised the presentation of tables to reduce redundancy and improve clarity. Table 1 now focuses on baseline characteristics, presenting continuous variables (means ± SD) alongside key categorical demographic, socioeconomic, behavioral, and nutritional variables, while retaining the continuous hematological parameters (hemoglobin, MCHC) to facilitate interpretation of the primary outcomes. Table 2 now focuses exclusively on hematological abnormalities, presenting prevalence data for erythrocyte, thrombocyte, and coagulation parameters, with all categorical abnormality data consolidated in this table to eliminate redundancy with Table 1. This restructuring eliminates redundancy while maintaining the comprehensiveness of the data presentation.

Comment 11: Discussion of Coagulation Parameters

The reviewer noted that the findings related to coagulation parameters (PT/APTT) were not sufficiently discussed. We have added a new subsection in the Discussion to address these findings. The new text reads: "An additional observation from our data was the higher prevalence of prolonged PT and APTT among smokers compared to non-smokers (54.2% vs. 42.5% for PT, p=0.012; 66.7% vs. 53.5% for APTT, p=0.005). While the primary focus of this study was on anemia, these findings warrant brief consideration. Chronic tobacco exposure is known to affect coagulation pathways through multiple mechanisms, including increased platelet activation, altered fibrinogen levels, and potential effects on vitamin K metabolism. However, given the cross-sectional design, we cannot determine whether these differences reflect direct biological effects of smoking or residual confounding by other factors. These findings highlight the complex, multi-system effects of smoking that may operate simultaneously in opposite directions across different physiological parameters."

Additional Technical Improvements

In addition to addressing the specific comments raised by the reviewers, we have made several technical improvements to the manuscript:

Figure Format Conversion

In accordance with PLOS ONE's technical requirements, we have converted all figures from PNG to TIF format. This conversion ensures that our figures meet the journal's specifications for high-resolution images suitable for publication. All figures (Figure 1, Figure 2, Figure 3, and Supplementary Figures S1 through S5) are now provided as TIF files with a minimum resolution of 300 DPI.

Table Revisions

We have revised all three main tables to improve clarity and reduce redundancy:

· Table 1 now focuses on baseline characteristics, presenting continuous variables (means ± SD) alongside key categorical demographic, socioeconomic, behavioral, and nutritional variables, while retaining the continuous hematological parameters (hemoglobin, MCHC) to facilitate interpretation

· Table 2 now focuses exclusively on hematological abnormalities, presenting prevalence data for erythrocyte, thrombocyte, and coagulation parameters, with all categorical abnormality data consolidated in this table to eliminate redundancy with Table 1

· Table 3 now presents full logistic regression models for both primary outcomes with all covariates (age, gender, BMI, university site), providing complete transparency of the adjusted associations

Supporting Information File Reorganization

We have reorganized and renamed the supporting information files for clarity and consistency. All files now follow a standardized naming convention, and we have verified that all files are accessible and functional.

Language and Style Refinements

Beyond the specific changes noted above, we have conducted a thorough review of the manuscript to ensure consistent use of neutral, objective language throughout. We have also verified that all statistical reporting follows standard conventions and that all abbreviations are properly defined.

Summary of Major Revisions

We have undertaken a comprehensive revision of the manuscript addressing all concerns raised by the reviewers and the Academic Editor. The key revisions are summarized below:

Methodological Revisions

· Reclassified smoking groups: former smokers (n=48) are now excluded from the primary analysis, with primary comparison between current smokers (n=144) and never-smokers (n=408)

· Revised sample size calculation using expected anemia prevalence, anticipated smoker/non-smoker ratio, and minimum detectable odds ratio

· Expanded Table 3 to present full multivariable models with all covariates

· Revised Table 1 and Table 2 to reduce redundancy and improve clarity, consolidating all categorical hematological abnormalities in Table 2 while retaining

continuous hematological parameters in Table 1 for ease of interpretation

· Added sensitivity analysis including former smokers to assess robustness

Interpretive Revisions

· Tempered causal language throughout all sections of the manuscript

· Reframed hypothesis statements with appropriate cautionary language

· Revised conclusion to use associative rather than definitive language

· Modified policy recommendations to reflect that the study informs rather than dictates priorities

Limitations and Discussion Revisions

· Expanded Limitations section to explicitly acknowledge unmeasured confounders (parasitic infections, menstrual blood loss, inflammation, micronutrient deficiencies)

· Added explicit acknowledgment of generalizability limitations

· Added acknowledgment of self-report limitations

· Added new subsection discussing coagulation parameter findings

· Clarified that mediation results are exploratory

Technical Revisions

· Converted all figures from PNG to TIF format

· Re-uploaded functional R scripts

· Anonymized participant data

· Corrected typographical errors

· Removed duplicate references

· Updated Data Availability Statement

· Unified ethics statement with approval number

Conclusion

We are sincerely grateful for the thorough and constructive reviews that have helped us substantially improve our manuscript. We believe that the revisions address all concerns raised by the reviewers and the Academic Editor. We have strengthened the methodological rigor, tempered our interpretations, expanded our discussion of limitations, improved the technical quality of our figures and tables, and enhanced the overall clarity of the manuscript.

We respectfully submit this revised manuscript and hope that it now meets the publication criteria of PLOS ONE. We thank you again for your time and expertise in reviewing our work, and we look forward to your favorable decision.

Respectfully submitted,

All Authors
